# Programmable nanomotor system responsively and chemotactically captures tumor associated antigens for enhanced *in situ* cancer vaccine

**DOI:** 10.1016/j.mtbio.2025.102543

**Published:** 2025-11-11

**Authors:** Panpan Song, Xiaoqing Han, Yanjing Wang, Xingbo Wang, Yaqing Kang, Jiao Yan, Haiyuan Zhang

**Affiliations:** a The First Affiliated Hospital of Guangzhou Medical University, Guangzhou Medical University, Guangzhou, 510120, China; b School of Biomedical Engineering, Guangzhou Medical University, Guangzhou, 511436, China; c Changchun Institute of Applied Chemistry, Chinese Academy of Sciences, Changchun, 130022, China; d School of Applied Chemistry and Engineering, University of Science and Technology of China, Hefei, 230026, China

**Keywords:** Nanomotors, Dendritic mesoporous silica nanoparticles, Outer membrane vesicles, TAA capture, Cancer immunotherapy

## Abstract

*In situ* cancer vaccines that utilize the body's own tumor-associated antigens (TAAs) to induce tumor-specific adaptive immune responses are emerging as a promising strategy in cancer therapy. However, the rapid clearance of TAAs due to innate immune system hinders the development of effective antitumor immunity. To address this challenge, we developed a nanomotor system (_D_DMSN@_M_OMV^PF^) as an *in situ* cancer vaccine capable of chemotactically capturing TAAs, significantly inhibiting the rapid clearance of TAAs and enhancing cancer immunotherapy. In response to acid tumor microenvironment, _D_DMSN@_M_OMV^PF^ exfoliated folate acid-attached, mitoxantrone-embedded bacterial outer membrane vesicle (OMV) fragments, which could be specifically taken up by tumor cells to induce immunogenic cell death (ICD) and release DNA-associated TAAs. Subsequently, the exposed DNase on _D_DMSN@_M_OMV^PF^ detected DNA gradient and propelled nanoparticles chemotactically capturing TAAs. *In vivo* results indicated that _D_DMSN@_M_OMV^PF^ suppressed both primary and distant tumors and elicited immune memory effects to prevent tumor recurrence.

## Highlights

• We develop DNase-powered nanomotor (_D_DMSN@_M_OMV^PF^) as an personalized *in situ* cancer vaccine.
• _D_DMSN@_M_OMV^PF^ can chemotactically capture and enrich TAAs under the guidance of the DNA gradient.
• _D_DMSN@_M_OMV^PF^ significantly inhibits the rapid clearance of TAAs by the innate immune system.
• _D_DMSN@_M_OMV^PF^ can suppress both primary tumor and distant tumor and prevent tumor recurrence.
• This *in situ* cancer vaccine exhibits active, programmable, sustained and personalized antitumor immunity.

## Graphical abstract

**Figure:**
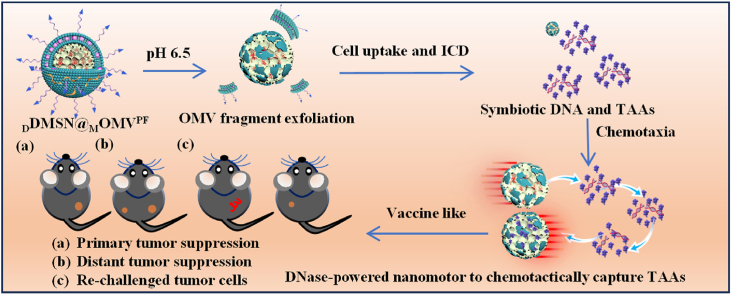

## 1 Introduction

Cancer vaccines have shown a considerable promise in eliminating tumors, establishing a long-term antitumor memory and minimizing non-specific or adverse reactions [1]. Although some traditional cancer vaccines have been developed using carefully selected, purified or prepared antigens alongside classical adjuvants, this strategy is often complex, costly, and effective only in a limited patient populations [2]. Recently, *in situ* cancer vaccine presents a promising alternative by directly attacking tumor cells and converting them into tumor-associated antigens (TAAs), thereby activating antigen-presenting cells (APCs) and T lymphocytes and initiating an immune response that ultimately eliminates tumor cells [[3], [4], [5]]. This strategy eliminates the need for patient-specific TAA identification and isolation, addressing the challenge of tumor heterogeneity and offering broader therapeutic potentials than traditional cancer vaccines [6,7]. The induction of immunogenic cell death (ICD) in tumor cells generates abundant TAAs, enabling the development of individualized *in situ* cancer vaccines that trigger tumor-specific immune responses [8]. This method provides a straightforward and efficient means of generating TAAs [9,10]. However, the efficacy of *in situ* cancer vaccines is often hindered by the innate immune clearance system and the immunosuppressive tumor microenvironment. Homeostatic processes rapidly degrade and eliminate the released TAAs, preventing sufficient recruitment of dendritic cells (DCs) for uptake, processing and presentation [11]. This results in ineffective absorption and recognition by APCs, ultimately hindering the immune response [12]. To address these challenges, a new *in situ* cancer vaccine strategy should be developed to efficiently capture and enrich the released TAAs while enhancing their delivery to APCs, thereby eliciting robust antitumor immunity.

Nanomotors, a class of nanoscale machines capable of converting various energy sources into mechanical movement, exhibit extensive diffusion and efficient cargo transport capabilities [13]. By leveraging the ubiquitous chemical gradients in biological scenarios, chemotactic nanomotors are able to implement self-navigation and self-targeting in complex biological microenvironment [14,15]. A series of precisely controllable nanomotors have been developed for applications in drug delivery [16,17], precision surgery [18,19], medical diagnosis [20] and environmental remediation [21,22], however, few nanomotors are explored for use in cancer vaccines. The motility of nanomotors improves their probability of encountering various gradients, thereby enhancing the TAA capture and enrichment. In the context of *in situ* cancer vaccination, a symbiotic relationship exists between TAAs and DNA, as the release of TAAs from apoptotic or dying tumor cells is associated with the release of a large amount of DNA. Considering the specific recognition between DNA and DNase, the subtle DNA gradient generated by apoptotic tumor cells can serve as a chemotactic attractant to guide the directional motion of DNase-incorporated nanomotors towards TAAs, facilitating the efficient enrichment of TAAs [23]. This DNase-powered nanomotor utilizes endogenous biofuel to generate a strong propulsive force, overcoming random Brownian motion and enabling effective self-propulsion [24]. Therefore, DNase-powered nanomotor shows great potential for capturing and enriching TAAs, making a significant contribution to cancer vaccine development.

**Fig. 1:**
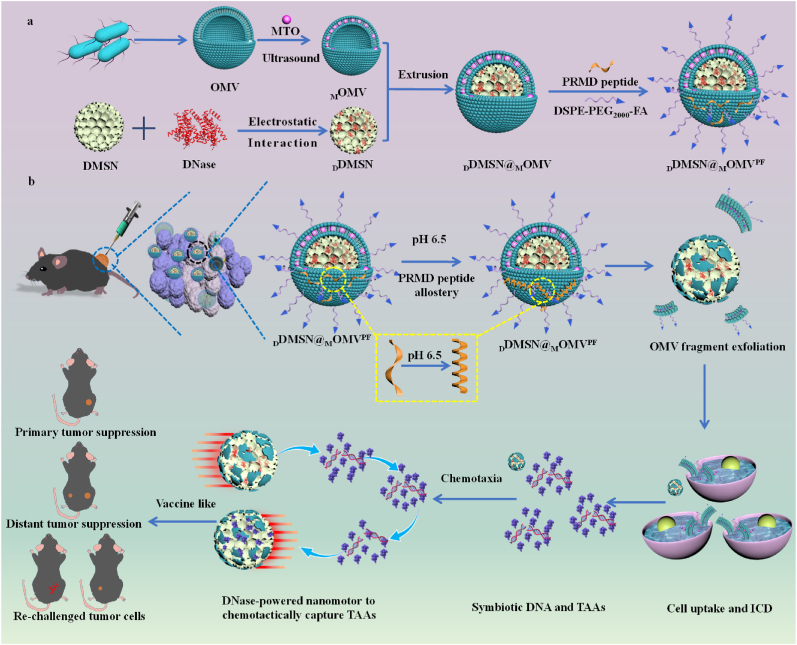
Schematic illustration of preparation and *in situ* cancer vaccine effects of _D_DMSN@_M_OMV^PF^. (a) Preparation of _D_DMSN@_M_OMV^PF^; (b) *In situ* cancer vaccine effects of _D_DMSN@_M_OMV^PF^; PRMD peptides on _D_DMSN@_M_OMV^PF^ undergo conformational changes in response to the acidic TME, releasing FA-attached, MTO-embedded OMV fragments and inducing ICD of tumor cells, thereby releasing symbiotic DNA and TAAs. Subsequently, the exposed DNase propelled _D_DMSN@_M_OMV^PF^ chemotactically migrating toward DNA, facilitating TAA capture and enrichment. _D_DMSN@_M_OMV^PF^, enriched with TAAs together with its intrinsic OMV, acts as an effective *in situ* cancer vaccine to improve DC maturation and T lymphocyte infiltration, suppressing both primary and distant tumors, along with the induction of long-term immune memory to prevent tumor recurrence.

In the present study, we developed a therapeutic nanomotor system to generate *in situ* cancer vaccine for triggering robust antitumor immune responses. This system effectively induces, captures and enriches TAAs, preventing their rapid clearance while leveraging bacterial outer membrane vesicles (OMVs) adjuvanticity for enhanced antigen presentation. OMV can activate a variety of TLR signaling pathways, this powerful immunogenicity makes OMV a potent adjuvant and delivery vehicle inducing DC maturation in cancer vaccines [25,26]. As shown in Fig. 1, mitoxantrone (MTO), a hydrophobic anticancer drug, was embedded into the hydrophobic phospholipid bilayer of OMVs derived from attenuated *Salmonella typhimurium* VNP20009, forming _M_OMV. DNase was immobilized onto the porous structures of dendritic mesoporous silica nanoparticles (DMSN) through electrostatic interaction, forming _D_DMSN. Through mechanical extrusion, _D_DMSN was encapsulated in _M_OMV, followed by functionalization with pH-responsive membrane-disruptive (PRMD) peptides (FLEHLIPHVIHGLVHAIHH-NH_2_) [27] through electrostatic interaction and distearoyl phosphatidylethanolamine-poly (ethylene glycol)-folate (DSPE-PEG_2000_-FA) through hydrophobic interaction to construct an *in situ* cancer vaccine (_D_DMSN@_M_OMV^PF^). Upon intratumorally injected into B1F1 tumor-bearing mice, PRMP peptides on _D_DMSN@_M_OMV^PF^ responded to acidic tumor microenvironment (TME) to compromise membrane integrity, causing membrane fragment exfoliation and DNase exposure. Due to the high expression of FA receptors in tumor cells, these exfoliated fragments attached with FA and embedded with MTO can be specifically taken up by the tumor cells, inducing ICD and facilitating the release of numerous bioactive molecules (such as small metabolites, nucleic acids and proteins) from dying tumor cells. Subsequently, the exposed DNase on _D_DMSN@_M_OMV^PF^ detected the DNA gradient from dying tumor cells and propelled the nanoparticles to chemotactically migrate toward DNA, facilitating TAA capture and enrichment through electrostatic interactions, as a result of formation of *in situ* cancer vaccine. Finally, TAA-enriched _D_DMSN@_M_OMV^PF^ was internalized by APC, eliciting a potent antitumor immune response. This *in situ* cancer vaccine strategy effectively induced ICD, promoted TAA release, capture and enrichment, enhanced antigen presentation, and stimulated robust antitumor T-cell responses, demonstrating promising therapeutic efficacy for cancer immunotherapy.

## 2 Experimental section

### 2.1 Materials

Tetraethyl orthosilicate (TEOS), cetyltrimethylammonium chloride (CTAC, ≥99.0 %), triethanolamine (TEA), 3-aminopropyltriethoxysilane (APTES, ≥80.0 %) were purchased from Aladdin (Shanghai, China). Cyclohexane was purchased from Xilong Chemical Co., Ltd (Shantou, China). DSPE-PEG_2000_-FA and MTO were obtained from Yuanye Bio-Technology (Shanghai, China). PRMD peptide was synthesized by Sangon Biotech (Shanghai, China). DSPE-PEG_2000_-Cy5.5 was purchased from MedChemExpress (Beijing, China).

### 2.2 Preparation of _D_DMSN nanomotors

DMSN NPs were synthesized following the previous method with some modifications [28]. 25 wt% CTAC solution (4 mL), 3.5 wt% TEA solution (1 mL) and ultrapure water (5 mL) were mixed and stirred (400 rpm) at 60 °C for 1 h. Then, 1.25 mL of TEOS and 8.75 mL of cyclohexane were added to the bottom of water phase. The mixture was stirred at 60 °C under a stirring speed of 400 rpm for 12 h. The solid samples were centrifuged at 10000 rpm for 30 min and washed with ethanol three times, the resulting products were annealed by calcination at 550 °C for 6 h to remove CTAC templates. Finally, the resulting products were functionalized with APTES, obtaining amino-functionalized nanoparticles (DMSN) for DNase-immobilized DMSN nanomotors (_D_DMSN). 0.27 g of the resulting products was dispersed in 40 mL of ethanol, followed by adding 800 μL of APTES. The mixture was vigorously stirred and refluxed at 80 °C overnight. Then DMSN was separated by centrifugation [29]. Then DNase solution (1 mL, 5 mg mL^−1^) (Sangon Biotech, Shanghai, China) was dropped in DMSN (10 mg mL^−1^) under 4 °C and stirred for 12 h. Finally, _D_DMSN was obtained through centrifugation at 8000 rpm for 10 min and washed with PBS buffer (pH 7.4) three times.

### 2.3 Preparation of _M_OMV

Attenuated Salmonella strain VNP20009 (ATCC 202165) was provided by the American Type Culture Collection. VNP20009 was cultured on Luria broth (LB) agar overnight at 37 °C, and then a single colony was inoculated into 50 mL of liquid LB medium at 37 °C for 12 h. The culture was diluted by fresh liquid LB medium in a ratio of 1:50 and further cultured at 37 °C for 3 h until the OD600 value of the medium reached approximately 1.0, indicating the logarithmic growth phase [30]. 500 mL of bacterial culture medium was centrifuged at 4000×*g* for 10 min to remove the bacteria and filtered through a 0.45 μm vacuum filter. The filtrate was then concentrated using an Amicon centrifugal filter with a molecular weight cutoff of 100 kDa (Millipore, USA). Then, the concentrate was centrifuged at 150,000×*g* for 2 h at 4 °C by using Optima L-80 XP ultra-centrifuge (Beckman Coulter) to obtain OMVs. The purified OMVs were obtained by ultracentrifugation at 4 °C and 150,000×*g* for 2 h using a sucrose density gradient, followed by removal of endotoxin using Detoxi-Gel Endotoxin Removing Columns [31](Thermo Scientific, Waltham, MA, USA). The OMVs were resuspended in PBS buffer and stored at −80 °C for further experiments. Total protein content was determined by BCA protein kit (EpiZyme, Shanghai, China). To prepare _M_OMV, 1 mL of MTO (100 μg mL^−1^) and 2 mL of OMVs (25 μg mL^−1^) were mixed together in PBS and incubated at 4 °C for 30 min, then subjected to sonication in a bath sonicator (VWR) for a duration of 30 s at a frequency of 35 kHz. Subsequently, they were chilled on ice for 60 s and then underwent another round of sonication in the bath sonicator for 30 s under identical conditions to form _M_OMV. Then, 100 kDa ultrafiltration membranes were utilized to remove free MTO.

### 2.4 Preparation of _D_DMSN@_M_OMV^PF^

For the preparation of _D_DMSN@_M_OMV^PF^, _D_DMSN suspension (1 mL, 1 mg mL^−1^) were mixed with _M_OMV (1 mL, 0.5 mg mL^−1^) under vortex. After that, the mixed solution was physically extruded through a 0.22 μm polycarbonate membrane using Avanti mini-extruder for seven passes to form _D_DMSN@_M_OMV [32]. _D_DMSN@_M_OMV were collected after centrifugation at 8000×*g* for 20 min and resuspended in PBS at 4 °C for the follow-up experiments. Then collected _D_DMSN@_M_OMV was co-incubation with 0.4 mg of PRMD peptide (FLEHLIPHVIHGLVHAIHH-NH_2_) and 1 mL of DSPE-PEG_2000_-FA (500 μg mL^−1^) to form _D_DMSN@_M_OMV^PF^. Similarly, DMSN or _D_DMSN suspension (1 mL, 1 mg mL^−1^) and OMV^PF^ or _M_OMV^PF^ (1 mL, 0.5 mg mL^−1^) were mixed to prepare DMSN@OMV^PF^, _D_DMSN@OMV^PF^ and DMSN@_M_OMV^PF^ NPs according to above method. The loading capacity of MTO was quantitatively evaluated by UV–vis absorption spectra at 610 nm using the equation: the loading capacity of MTO (%) = the weight of loaded MTO/the weight of whole nanoparticles × 100 %. For the preparation of DMSN@L^PF^, liposome (L) was prepared on the basis of thin-film hydration method [33]. Briefly, soybean phospholipid, cholesterol and DSPE-PEG2000 at a molar ratio of 4:4:1 was co-dissolved in 10 mL of chloroform, then the organic solvent was removed by a rotary evaporator in vacuum at 45°С, and a thin lipid film was formed on the surface of round-bottom flask. Finally, 10 mL of PBS (pH 7.4) was added to the thin lipid film, and the liposome was obtained by vortex. Subsequently, DMSN suspension (1 mL, 1 mg mL^−1^) and L (1 mL, 0.5 mg mL^−1^) were mixed to prepare DMSN@L^PF^ NPs according to above method.

### 2.5 Characterization of _D_DMSN@_M_OMV^PF^

The morphologies of nanoparticles were observed using a transmission electron microscope (TEM, JEOL). The specific surface area, pore volume, and pore size distribution of DMSN were determined by nitrogen adsorption-desorption isotherms using the Brunauer-Emmett-Teller (BET) method. The crystal structure of DMSN was analyzed by X-ray diffraction (XRD). The membrane protein on surface of extruded nanoparticles were characterized by SDS-PAGE gel [34]. All samples were heated to denature the proteins, and the same amount of protein was used for electrophoresis. After that, proteins were stained with Coomassie blue. Finally, image after decolorization. For OMV^PF^, _M_OMV^PF^ or L^PF^ fluorescence labeling, 50 μg of OMV^PF^, _M_OMV^PF^ or L^PF^ were mixed with the lipophilic green fluorescent dye DiO (10 μl, Molecular Probes; 1:100) for 30 min at 37 °C to achieve DiO-OMV^PF^, DiO-_M_OMV^PF^ or DiO-L^PF^. For DMSN fluorescence labelling, 20 mg of DMSN suspended in 20 mL of EtOH was mixed with 1 mg of Rhodamine B, and the resulting suspension was continuously stirred at 550 rpm at RT for 24 h. Rhodamine B-DMSN was collected through centrifugation and washed three times with EtOH and deionized water. Rhodamine B-DMSN^PF^ (1 mL, 0.1 mg mL^−1^) and DiO-OMV^PF^ (1 mL, 0.05 mg mL^−1^) were mixed and physically extruded through a 0.22 μm polycarbonate membrane using Avanti mini-extruder for seven passes, achieving effective encapsulation. In addition, Rhodamine B-DMSN or Rhodamine B-_D_DMSN suspension (1 mL, 0.1 mg mL^−1^) and DiO-OMV^PF^, DiO-_M_OMV^PF^ and DiO-L^PF^ (1 mL, 0.05 mg mL^−1^) were mixed to prepare fluorescence labeled DMSN@OMV^PF^, _D_DMSN@OMV^PF^, DMSN@_M_OMV^PF^ and DMSN@L^PF^ according to above method. Finally, fluorescence images were acquired on Leica SP8 confocal laser scanning microscope (Leica Microsystems, Mannheim, Germany). (DiO: λex = 483, λem = 501 nm; Rhodamine B: λex = 546, λem = 567).

### 2.6 MTO release

DMSN@_M_OMV^PF^ or _D_DMSN@_M_OMV^PF^ suspended in PBS (pH 7.4 and 6.5) was incubated in a shaker (100 rpm). At various time points, 200 μl of DMSN@_M_OMV^PF^ or _D_DMSN@_M_OMV^PF^ suspension was centrifuged at 10000 rpm for 20 min. The supernatant was collected and the absorbance at 610 nm was determined by SpectraMax M5 (Molecular Devices Corp, Los Angeles, CA USA).

### 2.7 Moving ability

To study the chemotaxis of nanomotors, a Slide-A-Lyzer mini dialysis device (20 kD MWCO, Thermo Scientific) was placed in one well of a 6-well cell culture plate. Then, 2.5 μmol L^−1^ DNA (100 μL) was added to the device, followed by the addition of 20 μL DMSN or _D_DMSN (10 μg L^−1^) to the well containing 1.6 mL of PBS. The nanomotors' trajectories were tracked by inverted optical microscope and analyzed by Image J. The MSD of DMSN or _D_DMSN was calculated by using the formula of MSD (Δt) = (xi(t + Δt)-xi(t))2 (i = 2), where x is a two-dimensional vector, i is an index to show x and y, and Δt represents the time interval [35]. De was obtained by fitting the MSD curves to Equation (1)

(1) $$MSD\;\left(\Delta t\right)=4D_{e}\Delta t$$

where D_e_ represents the effective diffusion coefficient and Δt represents the time interval [36].

### 2.8 Cell culture

The B16F1 cell line was obtained from American Type Culture Collection (ATCC) and cultured with high-glucose Dulbecco's modified Eagle's medium (DMEM) supplemented with fetal bovine serum (10 %), penicillin (100 units mL^−1^), streptomycin (100 mg mL^−1^) at 37 °C with 5 % CO2 [37].

### 2.9 Cellular uptake

The intracellular uptake of OMV fragments exfoliated from DMSN@OMV^PF^, _D_DMSN@OMV^PF^, DMSN@_M_OMV^PF^, _D_DMSN@_M_OMV^PF^ and DMSN@L^PF^ NPs in B16F1 cells was evaluated by Leica SP8 confocal laser scanning microscope (Leica Microsystems, Mannheim, Germany). Briefly, 1 × 10^4^ of B16F1 cells were seeded in Lab-Tek™ 8-well Chamber Slides (Nunc, Thermo Scientific) and incubated overnight. Meanwhile, DiO-modified DMSN@OMV^PF^, _D_DMSN@OMV^PF^, DMSN@_M_OMV^PF^, _D_DMSN@_M_OMV^PF^ or DMSN@L^PF^ (equal concentration 20 μg mL ^−1^ of DMSN) were incubated in DMEM medium (pH 6.5) for 24 h. Subsequently, the fragments containing DiO-modified OMV^PF^ or L^PF^ were collected via centrifugation. After removal of old medium in Lab-Tek™ 8-well Chamber Slides, the collected fragments were added and further incubation for 6 h. The cells were washed with PBS, fixed in parformaldehyde (4 % in PBS), and then the cell nuclei were stained with Hoechst 33258 (Beyotime Biotechnology) for 10 min while cell membrane was stained with wheat germ agglutinin-Alexa Fluor 594 fluorescent conjugate (WGA, Thermo Scientific). Finally, the cells were observed using a fluorescence microscope.

### 2.10 Cell viability, live/dead cell staining and cell apoptosis *in vitro*

B16F1 cells were incubated into 96-well plates (1 × 10^6^ cells/well) and incubated with DMSN@OMV^PF^, _D_DMSN@OMV^PF^, DMSN@_M_OMV^PF^, _D_DMSN@_M_OMV^PF^ and DMSN@L^PF^ NPs in different concentrations for 24 h at pH 6.5. The cell viability was determined using the CCK-8 assay [38,39]. For live/dead cell staining assay, the treated and untreated cells were stained with calcein-AM (1 μmol L^−1^) and PI (1 μmol L^−1^) for 15 min at room temperature in the dark, and the cellular fluorescence image was observed by inverted fluorescence microscope (Mshot M53, Micro-shot Technology Co. Ltd., Guangzhou, China). For cell apoptosis detection, the treated and untreated cells were stained with Annexin V-FITC/PI apoptosis detection kit (Meilunbio, Dalian, China), and apoptotic cells was analyzed by BD Accuri™ C6 flow cytometer.

### 2.11 Detection of ICD *in vitro*

To examine the CRT exposure, B16F1 cells were incubated in 24 well plates at a density of 5 × 10^4^ cells per well for 24 h, and DMSN@OMV^PF^, _D_DMSN@OMV^PF^, DMSN@_M_OMV^PF^, _D_DMSN@_M_OMV^PF^ or DMSN@L^PF^ (equal to 50 μg mL ^−1^ DMSN) was added for 12 h incubation at pH 6.5. Next, B16F1 cells were washed with cold PBS three times and fixed in 4 % paraformaldehyde for 10 min. After being washed with cold PBS three times, the cells were then incubated with CRT primary antibody (Beyotime Biotechnology, Shanghai, China) for 1 h, followed by washing with PBS three times and incubation with FITC-conjugated secondary antibody (Beyotime Biotechnology, Shanghai, China) for 30 min. Finally, the cells were stained with Hoechst 33258 (Beyotime Biotechnology, Shanghai, China) and analyzed by inverted fluorescence microscope (Mshot M53, Micro-shot Technology Co. Ltd., Guangzhou, China). To examine the HMGB1 localization, B16F1 cells were incubated in 24 well plates at a density of 5 × 10^4^ cells per well for 24 h. DMSN@OMV^PF^, _D_DMSN@OMV^PF^, DMSN@_M_OMV^PF^, _D_DMSN@_M_OMV^PF^ or DMSN@L^PF^ NPs (equal to 50 μg mL ^−1^ DMSN) was added to for 24 h at pH 6.5. Then, B16F1 cells were washed with cold PBS and fixed with 4 % paraformaldehyde for 20 min. Next, the cells were permeabilized with 0.1 % Triton X-100 for 10 min. Nonspecific binding sites were blocked by 5 % FBS in PBS for 30 min, followed by incubation with HMGB1 primary antibody (Beyotime Biotechnology, Shanghai, China) for 1 h, followed by washing with PBS three times and incubation with an PE-conjugated secondary antibody for 30 min. Finally, the cells were stained with Hoechst 33258 (Beyotime Biotechnology, Shanghai, China) and examined by fluorescence microscope. To examine the ATP release, B16F1 cells were incubated in 24 well plates at a density of 5 × 10^4^ cells per well for 24 h. DMSN@OMV^PF^, _D_DMSN@OMV^PF^, DMSN@_M_OMV^PF^, _D_DMSN@_M_OMV^PF^ or DMSN@L^PF^ NPs (equal to 50 μg mL ^−1^ DMSN) was added to for 24 h at pH 6.5. The supernatant was collected and detected using ATP detection kit (Solarbio, Beijing, China).

### 2.12 Antigen capture

B16F1 cells (1 × 10^4^ cell/well) were seeded in 96-wells plates and incubated for 24 h. Then, the cells were incubated with MTO for 24 h to induce the apoptosis of tumor cells. After that, _D_DMSN@_M_OMV^PF^ was added at pH 6.5, and optical microscope was used to record the movement of nanomotors at different positions. For antigen capture assays, B16F1 cells (4 × 10^4^ cell/well) were seeded in 24 wells plates and incubated for 24 h. Then, the cells were treated with DMSN@OMV^PF^, _D_DMSN@OMV^PF^, DMSN@_M_OMV^PF^, _D_DMSN@_M_OMV^PF^ or DMSN@L^PF^ NPs (equal to 50 μg mL ^−1^ DMSN) for 24 h at pH 6.5. The supernatant was collected through centrifugation at 1000 rpm for 5 min, and the TAA-captured NPs were separated from free TAAs through centrifugation at 8000 for 5 min. The amount of protein was determined using BCA assay. Subsequently, SDS-PAGE was carried out to verify that the NPs could capture the protein. The collected NPs were heated to denature the proteins, and the same amount of protein was used for electrophoresis. After that, proteins were stained with Coomassie blue and imaged after decolorization. Subsequently, the protein bands were enzymatically hydrolyzed, and the peptide segments were desalted, isolated, and identified by capillary liquid chromatography tandem mass spectrometry (LC-MS/MS). Raw data files were analyzed using Proteome Discoverer (PD) version 2.4 (Sequent HT, Thermo Scientific). Peak lists were searched against the Mus musculus Swissport database. Bioinformatic analysis of proteomic data was performed with the Majorbio Cloud platform (https://cloud.majorbio.com). Functional annotation of all identified proteins was performed using KEGG pathway (http://www.genome.jp/kegg/). Western blotting assay was used to further analyze the captured proteins. First of all, captured proteins were separated with 10 % SDS-PAGE and then transferred to a polyvinylidene fluoride transfer membrane. After incubation with GP100 antibody (1:1000; Abcam) or TRP-2 antibody (1:1000; Abcam) or OmpA antibody (1:1000; EpiGentek) overnight at 4 °C, the PVDF membranes were incubated with HRP-labeled goat anti-mouse lgG (H + L) (1:1000; Beyotime) for 2 h, and the brand was detected by chemiluminescence imaging system (Tanon 5200 Multi, Tanon, China).

### 2.13 Maturation of DC *in vitro*

Bone marrow dendritic cells (BMDCs) were obtained from the femur and tibia of C57BL/6 mice under sterile conditions. Then, BMDCs were cultured in RPMI 1640 medium supplemented with 10 % FBS, 100 U mL ^−1^ penicillin-streptomycin, 20 ng mL ^−1^ IL-4 and 20 ng mL ^−1^ GM-CSF. Meanwhile, B16F1 cells were incubated in 24 well plates (5 × 10^4^ cells/well) for 24 h. After that, the cells were treated with DMSN@OMV^PF^, _D_DMSN@OMV^PF^, DMSN@_M_OMV^PF^, _D_DMSN@_M_OMV^PF^ or DMSN@L^PF^ NPs (equal to 50 μg mL ^−1^ DMSN) for 24 h at pH 6.5. Then, the supernatant was collected through centrifugation at 1000 rpm for 5 min, and the TAA-captured NPs were collected from free TAAs through centrifugation at 8000 for 5 min and co-incubated with BMDCs for 24 h. Finally, BMDCs were harvested and stained with anti-CD11c-FITC (Biolegend, San Diego, CA, USA), anti-CD86-PE (Biolegend, San Diego, CA, USA), anti-CD80-APC (Biolegend, San Diego, CA, USA), and the matured DCs was analyzed by BD Accuri™ C6 flow cytometer, and the supernatant was collected to measure the levels of TNF-α and IL-6 by ELISA kits (BioLegend, San Diego, CA, USA).

### 2.14 Animals and antitumor efficiency

Female C57BL/6 mice were purchased from Beijing Vital River Experiment Animal Technology Co. Ltd, and kept in stainless steel cages with distilled water and sterilized food at about 20 °C and normal humidity. All animal procedures were approved by the Institutional Animal Care and Use Committee (IACUC) of the Animal Experiment Center of Guangzhou Medical University (Approval No: GY2024-621). We strictly adhered to the predetermined humane endpoints approved by our Institutional Animal Care and Use Committee throughout the study. These ethical thresholds included: tumor burden exceeding 10 % of the animal's original body weight, any tumor dimension surpassing 20 mm in diameter, development of ulceration or necrosis impairing normal function, significant body weight loss of 20–25 %, or any signs of severe distress. All experiments were terminated before any animal reached these endpoints to ensure ethical compliance and minimize suffering. 100 μl of B16F1 cells (1 × 10^6^ cells mL^−1^) were subcutaneously injected into the right frank of C57/BL6 mice to establish B16F1 tumor-bearing models. For biodistribution study, _D_DMSN@_M_OMV^PF^ (1 mg mL^−1^) was incubated with DSPE-PEG_2000_-Cy5.5 (100 μg mL^−1^) to form Cy5.5-labeled _D_DMSN@_M_OMV^PF^ to observe biodistribution of _D_DMSN@_M_OMV^PF^ in the body. For antitumor efficiency, when the volume of the primary tumor reached 100 mm^3^, the primary tumors were intratumorally injected with (a) PBS, (b) DMSN@OMV^PF^, (c) _D_DMSN@OMV^PF^, (d) DMSN@_M_OMV^PF^, (e) _D_DMSN@_M_OMV^PF^ and (f) DMSN@L^PF^ (equivalent to 50 mg kg ^−1^ of DMSN) on Day 0, Day 2 and Day 4, respectively. On Day 16, the mice were sacrificed and tumor tissues were removed for photography and H&E staining. During the treatments, the body weight and tumor volume were monitored every day. The tumor volume (mm^3^) was calculated as (width)^2^ × (length) × 1/2.

### 2.15 Immune responses *in vivo*

The B16F1 tumor-bearing mice were sacrificed, and tumor tissues and inguinal lymph nodes of each group were harvested on Day 5. The tumor tissues and inguinal lymph nodes were minced and filtered through a 300-mesh screen to get single cells, followed by washing with cold PBS. The cells were centrifuged at 1100 rpm for 5 min, resuspended with 100 μL of cold PBS, and labeled with fluorescent antibodies according to the manufacturer's protocols. For the analysis of CRT exposure on tumor cell surfaces, single-cell suspensions prepared from dissociated tumor tissues were incubated with a primary anti-CRT mouse monoclonal antibody (1:1000) at room temperature for 2 h. After centrifugation and washing with PBS, the cells were subsequently incubated with a FITC-conjugated goat anti-mouse IgG (H + L) secondary antibody for 1 h at room temperature in the dark. The percentage of CRT-positive cells was then quantified using a flow cytometer. The release level of HMGB1 in tumor tissues was detected using an ELISA kit. Briefly, tumor tissues were homogenized in cold lysis buffer, and the homogenates were centrifuged at 8000×*g* for 10 min. The resulting supernatants were collected, and the HMGB1 concentration was measured strictly according to the manufacturer's protocol. Then, the matured DCs (CD11c^+^CD80^+^CD86^+^) in inguinal lymph nodes and the tumor-infiltrating T lymphocytes CD8^+^ T cells (CD3^+^ CD8^+^) and CD4^+^ T cells (CD3^+^ CD4^+^) were evaluated by flow cytometry. For the levels of TNF-α and IFN-γ in tumor tissues, tumor tissues were homogenized in cold tissue lysis buffer (Beyotime, China) with ice-bath. The supernatants were obtained by centrifuging at 8000×*g* for 10 min, and then detected by ELISA kits (BioLegend, San Diego, CA, USA) according to the manufacturer's protocols.

### 2.16 *In vivo* biosafety analysis

To evaluate the *in vivo* safety of DMSN@OMV^PF^, _D_DMSN@OMV^PF^, DMSN@_M_OMV^PF^, _D_DMSN@_M_OMV^PF^ and DMSN@L^PF^ NPs, serum samples were harvested at the end of 16 days of treatments, ALT, AST, BUN, LDH and CRE were measured to evaluate the functions of liver and kidney according to the manufacture's instruction (Changchun Baisheng Medical Laboratory, China) using a semiautomatic biochemical analyzer. At the same time, the mice were sacrificed and major organs (heart, liver, spleen, lung, kidneys) were collected to H&E staining.

### 2.17 Abscopal effect

Abscopal effect of various NPs was investigated using C57BL/6 mice with bilateral tumor model [40]. Briefly, B16F1 cells were subcutaneously inoculated on the right flank (5 × 10^4^ cells/mouse) for the primary tumor growth, and after 4 days, 1 × 10^4^ cells per mouse were subcutaneously inoculated on the left flank for distant tumor growth. When the volume of the primary tumor reached 100 mm^3^, the primary tumor was treated with various NPs as described above, and the distal tumor was without any treatment. Tumor volume was measured every two days to evaluate the treatment effect. All mice were sacrificed on Day 16 and analyzed for immune cell infiltration. Distant tumor tissues and spleen were minced and filtered through a 300-mesh screen to obtain single cells, and helper T cell of CD4^+^ T cell (CD3^+^CD4^+^ T cells) and cytotoxic T cell of CD8^+^ T cell (CD3^+^CD8^+^ T cells) were stained by using anti-CD3-FITC (BioLegend, San Diego, CA, USA), anti-CD4-PE (BioLegend, San Diego, CA, USA) and anti-CD8-APC (BioLegend, San Diego, CA, USA) antibodies for flow cytometry analysis. Meanwhile, the serum in each group was also collected for measurement of INF-γ and TNF-α using ELISA kits (BioLegend, San Diego, CA, USA).

### 2.18 Evaluation of immune-memory effects

To construct a secondary B16F1 tumor inoculation model after surgical ablation or _D_DMSN@_M_OMV^PF^ treatment. Firstly, 5 × 10^4^ of B16F1 cells were subcutaneously inoculated in the right flank of C57BL/6 mice. When the tumor volume reached around 100 mm^3^, the mice were randomly divided into 6 groups (n = 10) and a) control group (removing the tumor by surgery) or intratumorally injected with b) DMSN@OMV^PF^, c) _D_DMSN@OMV^PF^, d) DMSN@_M_OMV^PF^, e) _D_DMSN@_M_OMV^PF^ and f) DMSN@L^PF^ on Day 0, 2 and 4. On Day 40, five mice of each group were sacrificed and their spleens were extracted to study the effector memory T cells [41]. The rest five mice were re-inoculated with 1 × 10^4^ of B16F1 cells on the left flanks on Day 40. The tumor volume was measured every other day until Day 64.

### 2.19 Statistical analysis

Statistical analysis was carried out by GraphPad Prism 6.0. All data were expressed as mean ± SD. The data of different groups were compared using One-way ANOVA analysis. The level of significance was defined at ∗p < 0.05, ∗∗p < 0.01, ∗∗∗p < 0.001, ∗∗∗∗p < 0.0001.

## 3 Results and discussion

### 3.1 Fabrication and characterization of _D_DMSN@_M_OMV^PF^

**Fig. 2:**
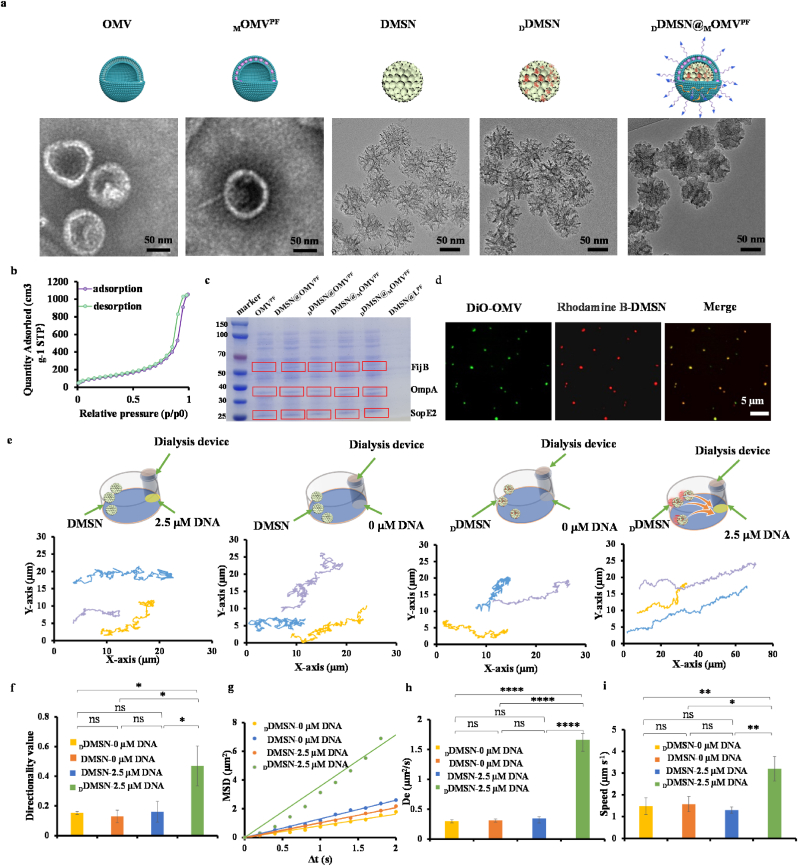
Characterization and analysis of the motion behavior of _D_DMSN@_M_OMV^PF^. (a) TEM image of OMV, _M_OMV^PF^, DMSN, _D_DMSN and _D_DMSN@_M_OMV^PF^; (b) N_2_ adsorption-desorption isotherms of DMSN; (c) SDS-PAGE analysis of DMSN@OMV^PF^, _D_DMSN@OMV^PF^, DMSN@_M_OMV^PF^, _D_DMSN@_M_OMV^PF^ and DMSN@L^PF^ NPs to the bands of FijB, OmpA and SopE2 derived from OMV; (d) CLSM images of _D_DMSN@_M_OMV^PF^ with OMV labeled by DiO (green) and DMSN by Rhodamine B (red); (e) Schematic illustration of chemotaxis movement and representative tracking trajectories of DNase-powered nanomotor in the absence or presence of DNase (n = 3); A Slide-A-Lyzer MINI dialysis device (20 kD MWCO) containing DNA (0 or 2.5 μmol L^−1^) was placed in one well containing 1.6 mL of PBS, followed by addition of DMSN or _D_DMSN, and the trajectories were recorded via inverted optical microscopy; (f) Directionality values; (g) Mean-squared displacement (MSD) plots; (h) De obtained by analyzing the MSD of DNase-powered nanomotor in the absence or presence of DNase; (i) Moving speeds of DMSN and _D_DMSN in the presence or absence of DNA. Moving speed is defined as v = s/t, where s is the travel distance in the time t. Data are presented as mean ± SD, the level of significance was defined at ∗*p* < 0.05, ∗∗*p* < 0.01, ∗∗∗*p* < 0.001, ∗∗∗∗*p* < 0.0001, ns: not significant (*p* > 0.05).

OMV was collected from the culture supernatants of attenuated *Salmonella typhimurium* [31,42]. Of note, an average of 20 μg OMV was harvested from 1 × 10^10^ attenuated *Salmonella typhimurium* colony-forming units (CFU). Transmission electron microscopy (TEM) images revealed the spherical morphology of OMVs with a primary size of 95.9 ± 4.9 nm (Fig. 2a). Dynamic light scattering (DLS) and zeta potential measurements further confirmed their hydrodynamic size of 109.8 ± 9.7 nm and zeta potential of −15.2 ± 0.7 mV in phosphate buffered saline (PBS) (Figs. S1 and S2). To facilitate the embedment of MTO into phospholipid bilayer of OMV, MTO was incubated with OMV under sonication, forming _M_OMV, which retained the spherical morphology with a primary size of 95.1 ± 3.4 nm (Fig. 2a), hydrodynamic size of 114.3 ± 3.8 nm (Fig. S1) and zeta potential of −16.9 ± 2.2 mV (Fig. S2). The loading capacity of MTO in _M_OMV was determined to be 10.1 % based on absorption measurement at 610 nm.

TEM image showed a homogeneous pore structure of DMSN with a primary size of 74.3 ± 1.8 nm (Fig. 2a), hydrodynamic size of 123.5 ± 3.8 nm (Fig. S1). X-ray diffraction (XRD) pattern of DMSN presented a characteristic diffraction peak centered on 23° (2θ) (Fig. S3), confirming its amorphous phase. Fig. 2b shows typical type-IV nitrogen adsorption/desorption isotherms, indicating a high density of mesopores. The Brunauer-Emmett-Teller (BET) analysis determined the specific surface area of DMSN as 396.8 m^2^ g^−1^ and the pore diameter as 7.8 nm.

Then, DNase was immobilized onto the surface of DMSN through electrostatic interactions to form _D_DMSN. TEM image confirmed _D_DMSN with a primary size of 78.1 ± 2.9 nm (Fig. 2a), hydrodynamic size of 130.0 ± 2.4 nm (Fig. S1). In addition, the zeta potential analysis showed a decrease from 20.1 ± 0.2 mV in DMSN to 5.5 ± 0.5 mV in _D_DMSN (Fig. S2), confirming the successful immobilization of DNase. The mass percentage of DNase in _D_DMSN was determined to be 9.8 % based on bicinchoninic acid (BCA) analysis.

To assemble the final nanomotor system, _D_DMSN was encapsulated with _M_OMV through mechanical extrusion, forming _D_DMSN@_M_OMV, which was further modified with PRMD peptides through electronic interaction and DSPE-PEG_2000_-FA through hydrophobic interaction to yield _D_DMSN@_M_OMV^PF^. For comparison, similar extrusion processes were used to prepare a range of control nanoparticles: OMV-encapsulated DMSN (DMSN@OMV), _M_OMV-encapsulated DMSN (DMSN@_M_OMV), OMV-encapsulated _D_DMSN (_D_DMSN@OMV) and liposome (L)-encapsulated DMSN (DMSN@L). The TEM images confirm the spherical morphology of the liposome with the hydrodynamic size of 124.0 ± 0.8 nm and zeta potential of −22.4 ± 0.2 mV (Figure S1, 2 and 4). These were subsequently modified by PRMD peptides and DSPE-PEG_2000_-FA to form DMSN@OMV^PF^, DMSN@_M_OMV^PF^, _D_DMSN@OMV^PF^ and DMSN@L^PF^, respectively (Fig. S4). As shown in Figs. S5 and S6, the hydrodynamic sizes of DMSN@OMV^PF^, DMSN@_M_OMV^PF^, _D_DMSN@OMV^PF^, _D_DMSN@_M_OMV^PF^ and DMSN@L^PF^ were 134.1 ± 4.1 nm, 139.7 ± 9.0 nm, 135.1 ± 6.8 nm, 136.6 ± 1.5 nm and 138.3 ± 4.2 nm, respectively, and their zeta potentials were 18.0 ± 0.2 mV, 17.1 ± 0.9 mV, 17.5 ± 0.6 mV, 17.6 ± 1.4 mV and −22.8 ± 1.3 mV, respectively. Sodium dodecyl sulfate polyacrylamide gel electrophoresis (SDS-PAGE) analysis identified the typical proteins associated with attenuated OMVs [43], such as flagellin (FijB), outer membrane protein A (OmpA) and invasion associated secretory effector protein (SopE2), on above various nanoparticles (Fig. 2c). At the same time, OMVs were labeled with DiO (green), while DMSN was labeled by Rhodamine B (red). Confocal laser scanning microscope (CLSM) images demonstrated the successful colocalization of DiO-labeled OMV and Rhodamine B-labeled DMSN, confirming the formation of DMSN@OMV^PF^, _D_DMSN@OMV^PF^, DMSN@_M_OMV^PF^, _D_DMSN@_M_OMV^PF^ and DMSN@L^PF^ (Fig. 2d and Fig. S7). Collectively, the above evidence indicates the successful construction of _D_DMSN@_M_OMV^PF^.

To demonstrate the morphological changes of DMSN@_M_OMV^PF^ and _D_DMSN@_M_OMV^PF^ at acidic environment, dynamic light scattering (DLS), zeta potential analysis, and transmission electron microscopy (TEM) were performed. As shown in Figure S5 and Figure S8a, after incubation at pH 6.5, the hydrodynamic diameters of both DMSN@_M_OMV^PF^ and _D_DMSN@_M_OMV^PF^ showed a decreasing trend. For DMSN@_M_OMV^PF^, the size changed from 139.7 ± 9.0 nm (pH 7.4) to 130.3 ± 4.1 nm (pH 6.5). For _D_DMSN@_M_OMV^PF^, the size changed from 136.6 ± 1.5 nm (pH 7.4) to 133.3 ± 1.0 nm (pH 6.5). As shown in Fig. S8b, after incubation at pH 6.5, the surface zeta potentials of both DMSN@_M_OMV^PF^ and _D_DMSN@_M_OMV^PF^ became less negative. These observations are consistent with the shedding of the OMV layer, which accounts for a substantial portion of the initial size and provides a negatively charged surface. To evaluate the surface zeta potential change of _D_DMSN@_M_OMV^PF^ under DNase-consumed conditions, _D_DMSN@_M_OMV^PF^ was incubated in PBS (pH 6.5) containing 2.5 μmol L^−1^ DNA for different time periods. As shown in Fig. S8c, the surface zeta potential of _D_DMSN@_M_OMV^PF^ reached −10.3 mV, 1.5 mV and 9.8 mV after 2, 4 and 6 h incubation, respectively, in the presence of 2.5 μmol L^−1^ DNA. Furthermore, TEM images revealed that the structural integrity of DMSN@_M_OMV^PF^ (Fig. S8d) and _D_DMSN@_M_OMV^PF^ (Fig. S8e) was compromised at pH 6.5 compared to that at pH 7.4. At the acidic pH, the histidine residues within the PRMR peptide become protonated, significantly increasing its net positive charge. This triggers their reorientation and insertion into the hydrophobic core of the OMV lipid bilayer, compromising the integrity and permeability of the OMV membrane. Furthermore, the drug release kinetics were evaluated over 48 h (Fig. S9). At pH 7.4, about 39.1 % and 38.4 % of MTO was released from DMSN@_M_OMV^PF^ and _D_DMSN@_M_OMV^PF^, respectively. In contrast, at pH 6.5, the drug release significantly increased, with nearly 78.4 % and 77.6 % of MTO release, respectively. In order to confirm the acid-responsive membrane rupture ability of the PRMD peptide, the release curve of DMSN@_M_OMV^F^ was further evaluated. As shown in Fig. S9, at pH 7.4, both DMSN@_M_OMV^PF^ and DMSN@_M_OMV^F^ exhibited slow and sustained release of MTO, indicating that the outer OMV membrane remained largely intact under neutral conditions. However, a notable difference was observed at pH 6.5. The release rate of MTO from DMSN@_M_OMV^PF^ increased significantly. In contrast, DMSN@_M_OMV^F^ showed a relatively lower release rate, comparable to that at pH 7.4. This suggests that under acidic conditions, the PRMR peptide is activated, disrupting the integrity of the OMV membrane and thereby facilitating the rapid release of MTO. To assess the motility of DMSN and _D_DMSN, their movement was tracked under an optical microscopy in the absence or presence of DNA (Fig. S10), and the corresponding motion trajectory was analyzed using ImageJ software (Fig. 2e). To quantify the directionality of the nanomotors, the ratio of linear displacement to travel distance is defined as the directionality value and calculated accordingly based on motion trajectory [24]. The closer this value is to 1, the higher the directionality of the nanomotors is. As shown in Fig. 2f, _D_DMSN that containing DNase has the higher directionality value in the presence of DNA, indicating that the _D_DMSN exhibits chemotactic motion toward DNA-rich regions. Mean-squared displacement (MSD) was calculated based on the tracked trajectories (Fig. 2g), and the corresponding effective diffusion coefficient (D_e_) was.

calculated based on MSD. In the absence of DNA, there was no noticeable difference in D_e_ between DMSN (0.3 μm^2^ s^−1^) and _D_DMSN (0.3 μm^2^ s^−1^). In the presence of DNA, D_e_ of DMSN remained at a low level (0.4 μm^2^ s^−1^), while D_e_ of _D_DMSN was significantly increased to 1.7 μm^2^ s^−1^ (Fig. 2h). Meanwhile, the average moving speed of the _D_DMSN was 3.2 μm s^−1^ in the presence of DNA, which was higher than DMSN (1.3 μm s^−1^) (Fig. 2i). These results confirm that DNase-mediated DNA hydrolysis enhances the motility of _D_DMSN, facilitating efficient movement and cargo transport.

### 3.2 Cell uptake, *in vitro* cytotoxicity and immunogenic cell death

**Fig. 3:**
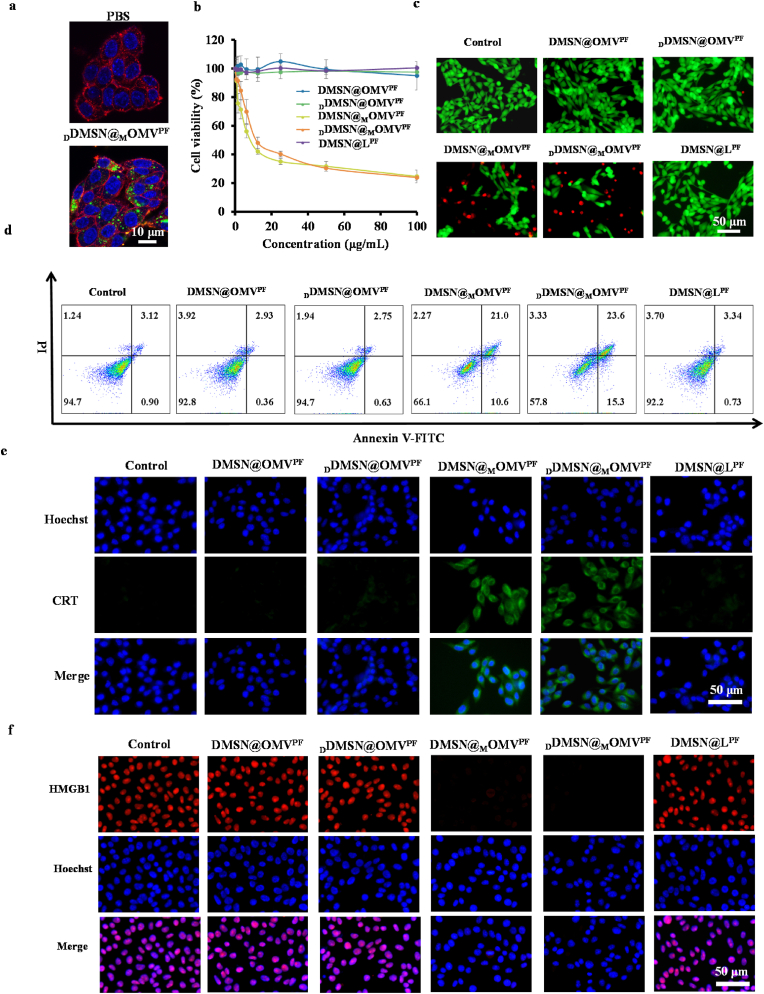
Cell uptake and ICD induction *in vitro*. (a) CLSM image analysis of DiO-labeled OMV fragment uptake by B16F1 cells treated with PBS or DiO-labeled _D_DMSN@_M_OMV^PF^; (b) CCK-8-based viability assessment of B16F1 cells treated with various NPs at different concentrations (equivalent to the content of DMSN) (n = 3); (c) Fluorescence microscopy images of live/dead B16F1 cells; live cells were stained with calcein-AM (green) while dead cells with PI (red); (d) Flow cytometry analysis of apoptosis of B16F1 cells based on Annexin V-FITC/PI staining; (e, f) Immunofluorescence images of CRT exposure (e) and HMGB1 release (f) of B16F1 cells; cells were incubated with CRT or HMGB1 primary antibody and FITC-conjugated or PE-conjugated secondary antibody successively. For (c)–(f), B16F1 cells were treated with DMSN@OMV^PF^, _D_DMSN@OMV^PF^, DMSN@_M_OMV^PF^, _D_DMSN@_M_OMV^PF^ and DMSN@L^PF^ NPs (equivalent to 20 μg mL^−1^ DMSN) for 24 h, respectively.

The cellular uptake of various fragments was investigated in B16F1 cells. CLSM images revealed intense green fluorescence in treated B16F1 cells (Fig. 3a and Fig. S11), suggesting the significant cellular uptake of OMV or L fragments, which is attributed to the presence of folate receptors (FRs) in melanoma cells [44].

Subsequently, the *in vitro* antitumor effect of various NPs was evaluated in B16F1 cells using CCK-8 assay. DMSN@OMV^PF^, _D_DMSN@OMV^PF^ and DMSN@L^PF^ NPs that lacked MTO showed minimal impact on cell viability (Fig. 3b), while DMSN@_M_OMV^PF^ and _D_DMSN@_M_OMV^PF^ that contained MTO effectively reduced cell viability, indicating their potent cytotoxic effects. The cytotoxicity results were further confirmed using calcein-AM/propidium iodide (PI) live/dead cell staining assay, showing consistent trend (Fig. 3c). Furthermore, apoptosis induction was analyzed by flow cytometry using Annexin V-FITC/PI staining. Fig. 3d shows both DMSN@_M_OMV^PF^ and _D_DMSN@_M_OMV^PF^ effectively induced apoptosis in B16F1 cells. The percentages of early and late apoptosis were 10.6 % and 21.0 %, respectively, for DMSN@_M_OMV^PF^, and 15.3 % and 23.6 % for _D_DMSN@_M_OMV^PF^. In contrast, DMSN@OMV, _D_DMSN@OMV^PF^ and DMSN@L^PF^ only induced weak apoptosis (0.4–0.9 % early apoptosis; 2.8–3.3 % late apoptosis). These results demonstrate DMSN@_M_OMV^PF^ and _D_DMSN@_M_OMV^PF^ induce substantial cell death due to MTT release.

To evaluate ICD induction, immunofluorescence staining was performed to detect CRT exposure and HMBG1 release in B16F1 cells treated with different NPs at pH 6.5. As shown in Fig. 3e and f, DMSN@_M_OMV^PF^ and _D_DMSN@_M_OMV^PF^ significantly increased CRT fluorescence and reduced HMGB1 fluorescence in B16F1 cells than DMSN@OMV^PF^, _D_DMSN@OMV^PF^ and DMSN@L^PF^, indicating effective ICD induction. These results were further corroborated by flow cytometry analysis, which confirmed increased CRT expression in B16F1 cells treated with DMSN@_M_OMV^PF^ and _D_DMSN@_M_OMV^PF^ (Fig. S12). In addition, the ATP release was quantified using an ATP content detection kit. B16F1 cells treated with DMSN@_M_OMV^PF^ and _D_DMSN@_M_OMV^PF^ exhibited significantly elevated extracellular ATP level compared to other formulations (Fig. S13). Collectively, these results confirm that DMSN@_M_OMV^PF^ and _D_DMSN@_M_OMV^PF^ efficiently trigger the release of ICD-associated DAMPs, highlighting their potential for immunotherapeutic applications.

### 3.3 Antigen capture and DC maturation induced by _D_DMSN@_M_OMV^PF^

**Fig. 4:**
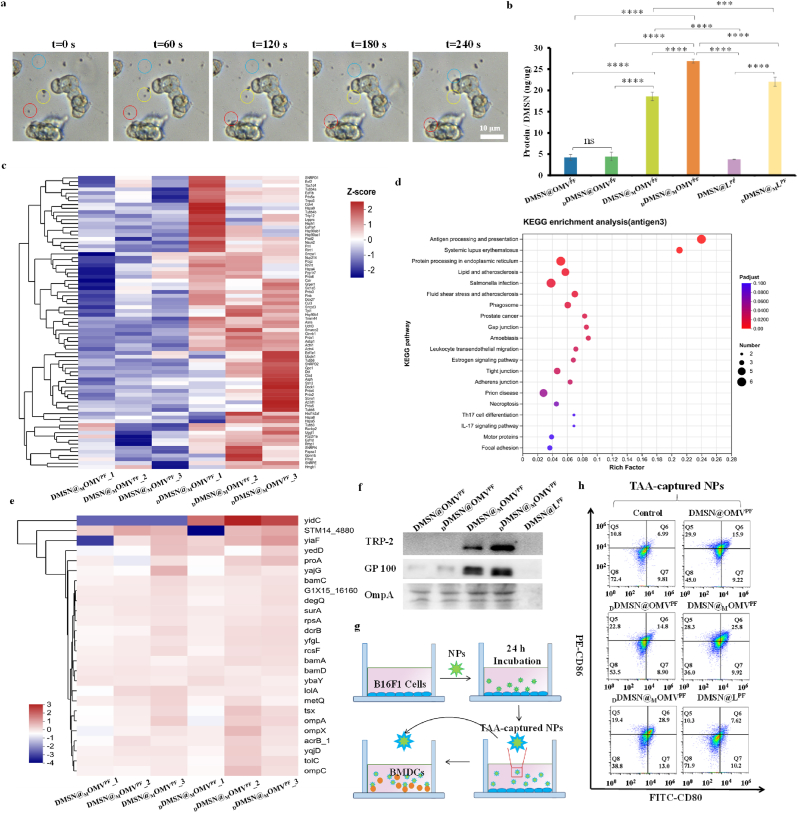
TAA capture and enrichment of B16F1 cells and DC maturation induced by _D_DMSN@_M_OMV^PF^. (a) Chemotactic nanomotor behavior of _D_DMSN@_M_OMV^PF^ gradually moving towards dying B16F1 cells at different times (n = 3); (b) BCA-based protein content analysis of various NPs after incubation with B16F1 cells (n = 3); (c) Heatmaps showing the relative abundance of TAAs and DAMPs captured by DMSN@_M_OMV^PF^ and _D_DMSN@_M_OMV^PF^; (d) KEGG enrichment analysis of the first 20 proteins; (e) Heatmaps showing the relative abundance of OMV proteins of DMSN@_M_OMV^PF^ and _D_DMSN@_M_OMV^PF^; (f) Western blot analysis of melanoma-associated antigen TRP-2 and GP100 and OMV-associated protein OmpA; (g) Schematic diagram of detecting DC maturation; (h) Flow cytometry analysis of DC maturation (CD11c^+^ CD80^+^ CD86^+^) induced by various formulations. Data are presented as mean ± SD, the level of significance was defined at ∗*p* < 0.05, ∗∗*p* < 0.01, ∗∗∗*p* < 0.001, ∗∗∗∗*p* < 0.0001. For (b) and (f), B16F1 cells were treated with DMSN@OMV^PF^, _D_DMSN@OMV^PF^, DMSN@_M_OMV^PF^, _D_DMSN@_M_OMV^PF^ and DMSN@L^PF^ NPs (equivalent to 20 μg mL^−1^ DMSN) for 24 h, respectively.

Following the induction of ICD, dying tumor cells release a large amount of TAAs, nucleic acids, and cell debris. DNase detects the subtle DNA gradient generated by dying tumor cells, and propels _D_DMSN dynamically moving toward DNA, facilitating the efficient and rapid capture of various TAA-associated proteins. As shown in Fig. 4a, when MTO induced B16F1 cells to undergo ICD and release subtle DNA gradient, the _D_DMSN exhibited chemotactic behavior toward the dying B16F1 cells. To examine the TAA capture ability of various NPs, B16F1 cells were incubated with DMSN@OMV^PF^, _D_DMSN@OMV^PF^, DMSN@_M_OMV^PF^, _D_DMSN@_M_OMV^PF^ or DMSN@L^PF^ for 24 h at pH 6.5, and the supernatant was collected and the TAA-captured NPs were separated from free TAAs by differential centrifugation for protein content analysis. SDS-PAGE gel analysis revealed more abundant protein bands in DMSN@_M_OMV^PF^ and _D_DMSN@_M_OMV^PF^ compared with DMSN@OMV^PF^, _D_DMSN@OMV^PF^ and DMSN@L^PF^ (Fig. S14). BCA quantification confirmed that the protein content in _D_DMSN@_M_OMV^PF^ (26.9 μg protein μg^−1^ DMSN) was significantly higher than those of DMSN@_M_OMV^PF^ (18.6 μg protein μg^−1^ DMSN), _D_DMSN@OMV^PF^ (4.4 protein μg^−1^ DMSN), DMSN@OMV^PF^ (4.2 protein μg^−1^ DMSN), DMSN@L^PF^ (3.8 protein μg^−1^ DMSN) and _D_DMSN@_M_L^PF^ (22.1 μg protein μg^−1^ DMSN) (Fig. 4b), indicating DNase-powered motility enhances TAA enrichment. Furthermore, we characterized the collected nanoparticles using TEM. As shown in Fig. S15, a distinct dense layer can be clearly observed on the surface of the nanoparticles after incubation with the lysate of MTO-treated tumor cells. This observed layer was attributed to the tumor-associated antigens (TAAs) and other proteins captured from the cell lysate. Next, to determine whether these captured proteins contained TAAs, proteomic analysis was conducted. We conducted a correlation analysis on the total protein samples captured, and the results showed that the correlation coefficients between biological replicates were all above 0.97, indicating high intra-group consistency and reliability of our data. (Fig. S16). As shown in Fig. 4c, DMSN@_M_OMV^PF^ and _D_DMSN@_M_OMV^PF^ successfully captured many TAAs and DAMPs caused by ICD, with _D_DMSN@_M_OMV^PF^ showing a higher abundance than DMSN@_M_OMV^PF^ due to DNase-powered motor capacity. Among them, 36 tumor neoantigens that have already been reported [45] to activate tumor-specific immune responses were captured by DMSN@_M_OMV^PF^ and.

_D_DMSN@_M_OMV^PF^ (Fig. S17). Further, Kyoto Encyclopedia of Genes and Genomes (KEGG) pathway enrichment analysis and abundance statistics indicated that these captured proteins were involved in antigen processing and presentation (Fig. 4d). Besides, OMV-derived proteins remained present on the surface of DMSN@_M_OMV^PF^ and _D_DMSN@_M_OMV^PF^ (Fig. 4e), facilitating the synergistic interaction between antigens and adjuvants within the local microenvironment and effectively forming an *in situ* cancer vaccine. Moreover, western blot analysis (Fig. 4f) further revealed the highest contents of GP100 (TAA), TRP-2 (TAA) and OmpA (bacteria protein) present on _D_DMSN@_M_OMV^PF^ among all the NPs, ensuring the co-delivery of TAAs and adjuvant to DCs for exerting *in situ* cancer vaccine-like effect.

To evaluate DC maturation, bone marrow-derived DCs (BMDCs) from C57BL/6 mice were treated with above TAA-captured DMSN@OMV^PF^, _D_DMSN@OMV^PF^, DMSN@_M_OMV^PF^, _D_DMSN@_M_OMV^PF^ or DMSN@L^PF^ NPs for 24 h (Fig. 4g). Fig. 4h and Fig. S18 shows that DMSN@_M_OMV^PF^ and _D_DMSN@_M_OMV^PF^ more effectively stimulated DC maturation than DMSN@OMV^PF^, _D_DMSN@OMV^PF^ and DMSN@L^PF^, with _D_DMSN@_M_OMV^PF^ exhibiting the more performance than DMSN@_M_OMV^PF^. To further evaluate the stimulation of BMDCs, cytokine secretion was measured using ELISA (Fig. S19). _D_DMSN@_M_OMV^PF^ triggered the highest secretions of IL-6 and TNF-α among all the NPs, consistent with its superior ability to stimulate DC maturation. The substantial enhancement of DC maturation in the _D_DMSN@_M_OMV^PF^ group highlights its strong potential in stimulation of DC maturation. Taken together, _D_DMSN@_M_OMV^PF^ can be powered by DNase to actively capture TAAs, leading to effective BMDC maturation and cytokine secretion. This process has the potential to activate both innate and adaptive immune responses, highlighting its promise as an *in situ* cancer vaccine strategy.

### 3.4 *In vivo* ICD induction, DC maturation, T cells immune response and antitumor effect

**Fig. 5:**
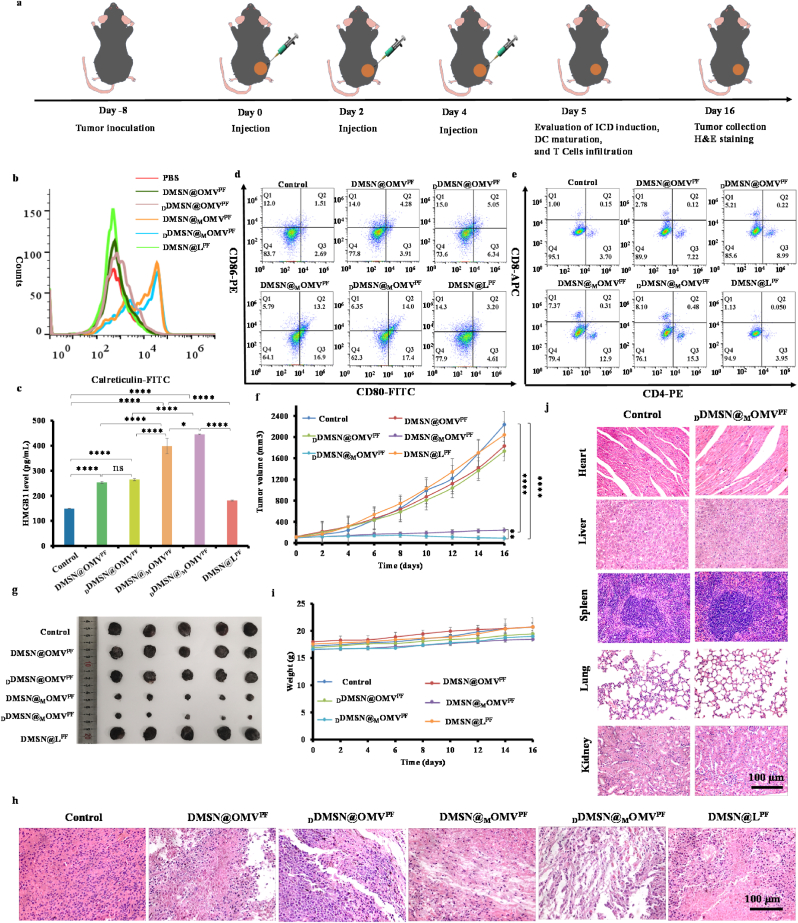
Antitumor immunity response and tumor growth inhibition in B16F1 tumor-bearing C57BL/6 mice. (a) Schematic illustration of B16F1 tumor-bearing mice primary model establishment and treatment procedures *in vivo*; (b) CRT expression on cell surface in tumor tissues of mice analyzed by flow cytometry; (c) HMGB1 levels in the isolated tumor extracts determined by ELISA (n = 3); (d) Proportions of mature DCs (CD11c^+^ CD80^+^ CD86^+^) in lymph nodes analyzed by flow cytometry; (e) Infiltration proportions of CD8^+^ T lymphocytes (CD3^+^ CD8^+^) and CD4^+^ T Lymphocyte (CD3^+^ CD4^+^) in tumor tissues analyzed by flow cytometry; (f) Tumor growth curves; (g) Images of tumors at the end of treatment (n = 5); (h) H&E staining images of tumor tissues at the end of treatment; (i) Body weight of mice during the treatment period; (j) H&E staining of major organs of mice after treated with PBS and _D_DMSN@_M_OMV^PF^ NPs for 16 days. For (b) to (e), B16F1 tumor-bearing C57BL/6 mice were treated with various NPs for 5 days (n = 5); For (F) to (J), mice were accordingly treated for 16 days (n = 5). Data are presented as mean ± SD, the level of significance was defined at ∗*p* < 0.05, ∗∗*p* < 0.01, ∗∗∗*p* < 0.001, ∗∗∗∗*p* < 0.0001.

Investigating the systemic biodistribution of nanotheranostic agents is essential for their potential biomedical applications. In vivo, fluorescence imaging with the IVIS spectrum system was conducted following the intratumorally administration of Cy5.5-labeled _D_DMSN@_M_OMV^PF^ at predefined time points (0, 2, 6, 12, 24, and 48 h). As shown in Fig. S20, the tumors exhibited a time-dependent increase in Cy5.5 fluorescence signal. Following the injection of _D_DMSN@_M_OMV^PF^ for 48 h, mice were sacrificed, and ex vivo fluorescence imaging of the primary organs (heart, liver, spleen, lung, and kidney) and the tumor was performed. The results revealed that the fluorescence intensity was notably higher in the tumor tissue at 48 h post-injection compared with other tissues and suggested that _D_DMSN@_M_OMV^PF^ nanoparticles demonstrated the prolonged retention (Fig. S21). Immunogenicity arises from two key factors, antigenicity and adjuvanticity. While antigenicity is necessary, it is insufficient to elicit an effective adaptive immune response. In the absence of co-stimulatory signals (such as adjuvants), the presentation of TAAs to T cells generally results in T cells anergy, contributing to peripheral tolerance [46]. Therefore, in view of co-delivery of antigens and adjuvants to exert excellent ability of enhancing tumor cell killing and promoting DC maturation at the cellular level, the *in vivo* vaccine-like immunomodulatory function of _D_DMSN@_M_OMV^PF^ was further studied in B16F1 tumor-bearing C57/BL6 mice model. When the tumor volume reached about 100 mm^3^, mice were divided into six groups (n = 5) and intratumorally injected with PBS, DMSN@OMV^PF^, _D_DMSN@OMV^PF^, DMSN@_M_OMV^PF^, _D_DMSN@_M_OMV^PF^ and DMSN@L^PF^ NPs, respectively. After 5 days of treatment, the tumors were collected from each group and ICD induction was investigated through assessment of CRT and HMGB1 levels using flow cytometry and ELISA, respectively (Fig. 5a). As shown in Fig. 5b and c, _D_DMSN@_M_OMV^PF^ triggered the highest levels of CRT exposure and HMGB1 release, followed by DMSN@_M_OMV^PF^, _D_DMSN@OMV^PF^, DMSN@OMV^PF^ and DMSN@L^PF^, consistent with *in vitro* results.

DC maturation promotes the binding of major histocompatibility complex (MHC) peptides to the T-cell receptor (TCR), which activates T cells [47]. The ability of _D_DMSN@_M_OMV^PF^ to recruit and activate DCs was assessed by measurement of mature DCs (CD11c^+^ CD80^+^ CD86^+^) in tumor-draining lymph nodes using flow cytometry (Fig. 5d and Fig. S22). After 5 days of treatment, the proportions of mature DCs in _D_DMSN@_M_OMV^PF^, DMSN@_M_OMV^PF^, _D_DMSN@OMV^PF^, DMSN@OMV^PF^, DMSN@L^PF^ NPs and PBS groups were 14.0, 13.2, 5.1, 4.3, 1.4 and 1.5 %, respectively. Notably, the maturation rate of DCs in _D_DMSN@_M_OMV^PF^ group was about 9.3-fold higher than that in PBS group, and 1.1-fold and 2.7-fold higher than those in DMSN@_M_OMV^PF^ and _D_DMSN@OMV^PF^ groups, respectively, confirming that the _D_DMSN@_M_OMV^PF^ boosted an effective immune response. The result suggests that the enriched TAAs and the co-existing OMV adjuvant of _D_DMSN@_M_OMV^PF^ elicit potent antitumor immune responses.

Following antigen and adjuvant stimulation, iDC matures and stimulates the proliferation and activation of naive T cells. CD8^+^ T cells play an important role in directly killing tumor cells and activating anti-tumor immune response [48], while CD4^+^ T cells are essential for regulating adaptive immunity [49]. Therefore, the populations of tumor-infiltrating CD8^+^ and CD4^+^ T cells were examined by flow cytometry after 5 days of treatment. As shown in Fig. 5e and Fig. S23, the infiltration of CD8^+^ and CD4^+^ T cells in _D_DMSN@_M_OMV^PF^ group reached 8.1 and 15.3 %, respectively, significantly surpassing those in DMSN@_M_OMV^PF^ (7.4 and 12.9 %), _D_DMSN@OMV^PF^ (5.2 and 9.0 %), DMSN@OMV^PF^ (2.8 and 7.2 %), DMSN@L^PF^ (1.1 and 4.0 %) and PBS groups (1.0 and 3.7 %). The enhanced T cell infiltration in _D_DMSN@_M_OMV^PF^ group is attributed to improved TAA capture and OMV co-delivery, which collectively facilitate antigen presentation and T cells activation. In addition, Elisa results (Fig. S24) further demonstrated that the levels of proinflammatory cytokines (such as TNF-α and IFN-γ) in _D_DMSN@_M_OMV^PF^ group were also significantly higher than those in DMSN@_M_OMV^PF^, _D_DMSN@OMV^PF^, DMSN@OMV^PF^, DMSN@L^PF^ and PBS groups, further supporting its ability to potentiate antitumor immune response. Various subtypes of IgG antibodies exhibit distinct activities, with IgG1 and IgG2a serve as indicators of humoral and cellular immunity, respectively. Therefore, the levels of IgG1 and IgG2a in the serum of treated mice were analyzed by ELISA (Fig. S25). The results revealed a significantly higher ratio of IgG2a to IgG1 (IgG2a/IgG1 > 1) in the _D_DMSN@_M_OMV^PF^ group compared to the control groups. The distinct antibody profile showing an IgG2a/IgG1 ratio above 1 establishes a predominant Th1-type immune response. These findings prove our nanomotor vaccine platform successfully drives Th1-skewed immunity which is essential for effective anticancer protection. Therefore, intratumorally injected _D_DMSN@_M_OMV^PF^ could release TAAs by ICD and efficiently capture them together with OMV to promote DC maturation and enhance T cells infiltration, resulting in a robust antigen-specific immune response.

Subsequently, B16F1 tumor-bearing C57/BL6 mice was further used to assess the *in vivo* therapeutic efficacy of _D_DMSN@_M_OMV^PF^. As shown in Fig. 5a, when the tumor volume reached 100 mm^3^, mice were also divided into six groups (n = 5) and intratumorally injected with PBS, DMSN@OMV^PF^, _D_DMSN@OMV^PF^, DMSN@_M_OMV^PF^, _D_DMSN@_M_OMV^PF^ and DMSN@L^PF^ NPs, respectively, on Day 0, Day 2 and Day 4. After 16 days of treatment, tumor growth was inhibited in DMSN@OMV^PF^, _D_DMSN@OMV^PF^, DMSN@_M_OMV^PF^ and _D_DMSN@_M_OMV^PF^ groups compared with PBS and DMSN@L^PF^ groups, however, the inhibition in DMSN@OMV^PF^ NPs and _D_DMSN@OMV^PF^ groups was less pronounced than that in DMSN@_M_OMV^PF^ and _D_DMSN@_M_OMV^PF^ groups, likely due to the lack of TAA enrichment (Fig. 5f and g). Notably, _D_DMSN@_M_OMV^PF^ exhibited superior tumor suppression compared to DMSN@_M_OMV^PF^ (p < 0.01), underscoring the critical contribution of the DNase-powered nanomotor to the therapeutic outcome by enhancing antigen capture and enrichment. At the end of 16 days of treatment, further histological analysis through hematoxylin and eosin (H&E) staining of tumor tissue sections revealed the most severe damage occurring in _D_DMSN@_M_OMV^PF^ group (Fig. 5h). Moreover, the assessment of apoptosis and proliferation of tumor cells was conducted using terminal-deoxynucleotidyl transferase-mediated nick end labeling (TUNEL) analysis and immunohistochemical staining with Ki-67 protein. As shown in Fig. S26, a noticeable induction of apoptosis following treatment with the DMSN@OMV^PF^ and _D_DMSN@_M_OMV^PF^ was demonstrated. Particularly, _D_DMSN@_M_OMV^PF^ exhibited significantly higher rates of apoptosis compared to DMSN@_M_OMV^PF^_, D_DMSN@OMV^PF^, DMSN@OMV^PF^, DMSN@L^PF^ and PBS. On the contrary, there were very few positive cells in Ki-67 in the _D_DMSN@_M_OMV^PF^, and the proliferation of tumor cells was inhibited. These proved that _D_DMSN@_M_OMV^PF^ can inhibit the proliferation and induce apoptosis of tumor cells. These results suggested that the extensive cancer cell death induced by _D_DMSN@_M_OMV^PF^ resulted from the synergistic effects of DNase-powered TAAs enrichment and the co-existing OMV adjuvant. Taken together, _D_DMSN@_M_OMV^PF^ effectively induced ICD within the tumor immunosuppressive microenvironment and facilitated DC maturation and subsequent CD4^+^ T and CD8^+^ T cell infiltration, which not only significantly inhibits tumor growth but also effectively stimulates the host immune system to fight cancer, suggesting its excellent efficacy in antitumor immunotherapy.

### 3.5 Biosafety assessment

To evaluate the *in vivo* biocompatibility of _D_DMSN@_M_OMV^PF^, body weight monitoring, histopathological evaluation and serum biochemistry analyses were performed. As shown in Fig. 5i, the body weights of the B16F1 tumor-bearing mice in all the groups remained stable throughout the treatment. H&E staining showed no inflammation or necrosis in the major organs (heart, liver, spleen, lung and kidney) of mice after treatment with various formulations (Fig. 5j and Fig. S27). Furthermore, hepatic and renal function parameters, including alanine aminotransferase (ALT), aspartate aminotransferase (AST), creatinine (CREA) and urea, remained within normal range across all groups without any abnormality (Fig. S28). These indicate that the OMV modified with DSPE-PEG_2000_-FA can mask dangerous surface signals and further demonstrate the excellent biocompatibility of these OMV^PF^-coated DMSN NPs.

### 3.6 Abscopal antitumor effect and immune responses

**Fig. 6:**
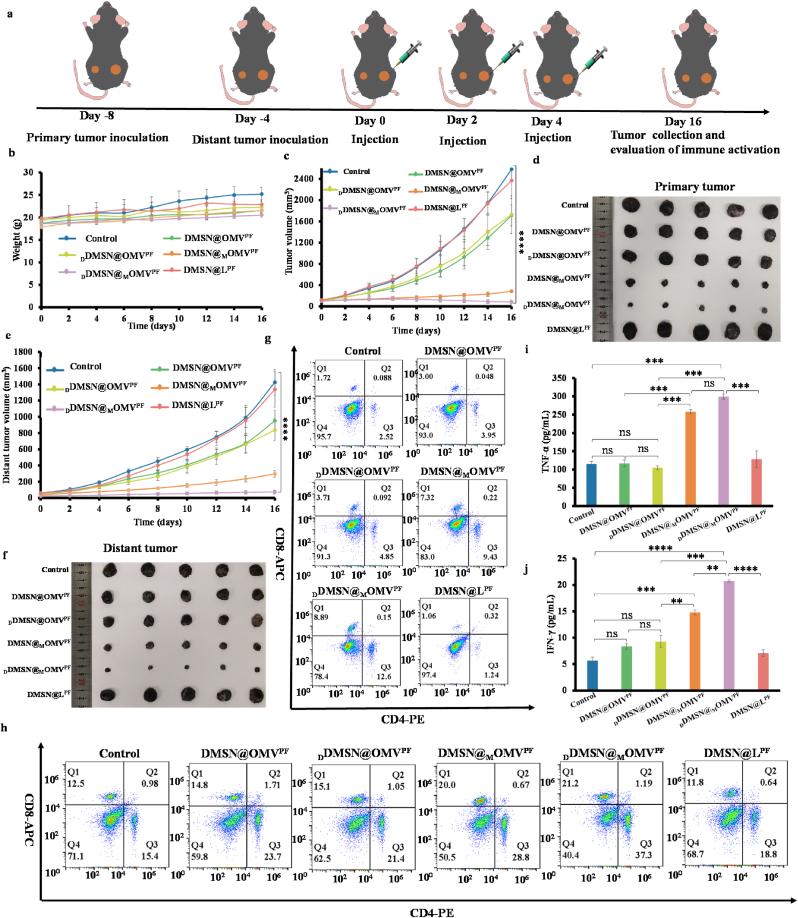
Distal tumor growth inhibition and antitumor immunity response. (a) Schematic illustration of B16F1 tumor-bearing mice bilateral model (primary tumor and distal tumor) establishment and treatment procedures *in vivo*; (b) Body weight of mice during various treatments; (c) Primary tumor growth curves (n = 5); (d) Primary tumor images at the end of treatment (n = 5); (e) Distal tumor growth curves (n = 5); (f) Distant tumor images at the end of treatment (n = 5); (g, h) Infiltration proportions of CD8^+^ (CD3^+^ CD8^+^) and CD4^+^ T lymphocytes (CD3^+^ CD4^+^) in distant tumors (g) and spleens (h) at the end of treatments, as analyzed by flow cytometry; (i, j) IFN-γ (i) and TNF-α (j) levels in serum at the end of treatments of were measured by ELISA after various treatments (n = 5). For (b) to (h), primary tumor was treated with various NPs on day 0,2,4, while no treatment was given to the distal tumors. Data are presented as mean ± SD, the level of significance was defined at ∗*p* < 0.05, ∗∗*p* < 0.01, ∗∗∗*p* < 0.001, ∗∗∗∗*p* < 0.0001.

To evaluate the robust vaccine-like immunological function of _D_DMSN@_M_OMV^PF^, a b ilateral tumor model was established to study the antitumor efficacy and abscopal effect of orthotopic vaccine *in vivo* [50]. When the primary tumor volume reached 100 mm^3^, mice were divided into six groups and received intratumoral injections of (a) PBS, (b) DMSN@OMV^PF^, (c) _D_DMSN@OMV^PF^, (d) DMSN@_M_OMV^PF^, (e) _D_DMSN@_M_OMV^PF^ or (f) DMSN@L ^PF^ on Day 0, 2 and 4 (Fig. 6a). As shown in Fig. 6b, no significant change was observed in the body weight of mice across the different groups over 16 days, indicating the excellent biocompatibility of these nanoparticles. Tumor growth in both primary and distant sites was monitored (Fig. 6c, d, 6e and 6f). The growth trend of primary tumors paralleled previous results (Fig. 5f and g), while the distal tumors were most effectively suppressed in the _D_DMSN@_M_OMV^PF^ group. Tumor-infiltrating CD8^+^ and CD4^+^ T cells from distant tumors were analyzed on Day 16 post-treatment. The proportions of CD8^+^ and CD4^+^ T cells were.

much higher in _D_DMSN@_M_OMV^PF^ group compared to other groups (Fig. 6g and Fig. S29). Furthermore, to demonstrate systemic immune activation, cytokine secretion in serum and CD8^+^ and CD4^+^ T cell proliferation in the spleen were also detected by ELISA and flow cytometry, respectively. Among all groups, _D_DMSN@_M_OMV^PF^ treatment induced the highest frequencies of CD4^+^ and CD8^+^ T cells in spleen (Fig. 6h and Fig. S29), along with the highest secretion levels of TNF-α (Fig. 6i) and IFN-γ (Fig. 6j) in serum. These results demonstrate that _D_DMSN@_M_OMV^PF^ can effectively suppress distant tumor growth through activating a systemic antitumor immune response.

### 3.7 Immune-memory effects

**Fig. 7:**
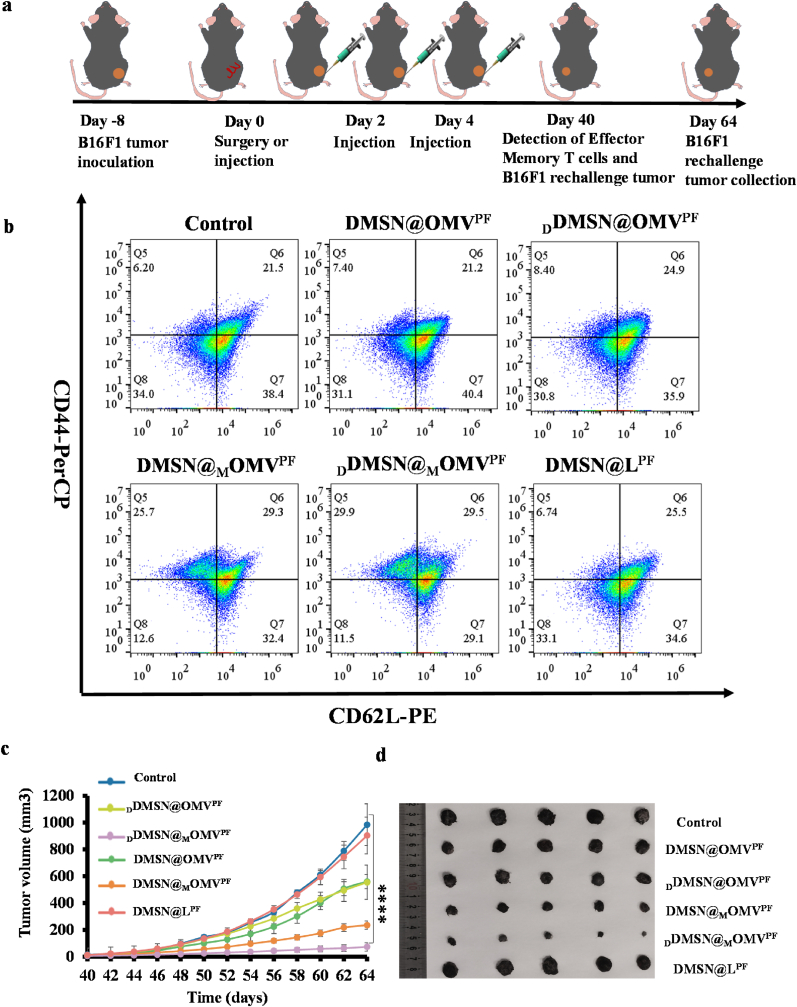
Long-term immune memory analysis. (a) Schematic illustration of B16F1 tumor-bearing mice tumor rechallenge model establishment and treatment procedures *in vivo*; (b) Flow cytometric quantification of proportions of effector memory T cells (CD3^+^ CD8^+^ CD44^+^ CD62L^−^) and central memory T cells (CD3^+^ CD8^+^ CD44^+^ CD62L^+^) expressed by splenic lymphocytes after immunizations; (c) Growth curves of isolated tumors at the end of treatment of recurrent tumors in B16F1 tumor-bearing C57BL/6 mice after different treatments (n = 5); (d) Images of recurrent tumors at the end of various treatments (n = 5). Data are presented as mean ± SD, the level of significance was defined at ∗*p* < 0.05, ∗∗*p* < 0.01, ∗∗∗*p* < 0.001, ∗∗∗∗*p* < 0.0001.

The generation of immune memory is essential for long-term tumor control and recurrence prevention. To evaluate whether _D_DMSN@_M_OMV^PF^ induced durable antitumor immunity, we further established a tumor rechallenge model. When the primary tumor volume reached 100 mm^3^, tumor clearance through surgical resection or intratumorally injected with DMSN@OMV^PF^, _D_DMSN@OMV^PF^, DMSN@_M_OMV^PF^, _D_DMSN@_M_OMV^PF^ and DMSN@L^PF^ treatment. Then, the left flank of the primary tumor model was inoculated with tumor cells as a rechallenge tumor model on day 40 (Fig. 7a). Effector memory T cells (CD3^+^ CD8^+^ CD44^+^ CD62L^−^) in splenocytes from primary B16F1 tumor-bearing C57/BL6 mice model were examined on Day 40 post-treatment. The percentages of CD44^+^ CD62L^−^ effector memory T cells were 6.2, 7.4, 8.4, 25.7, 29.9 and 6.7 % in PBS, DMSN@OMV^PF^, _D_DMSN@OMV^PF^, DMSN@_M_OMV^PF^, _D_DMSN@_M_OMV^PF^ and DMSN@L^PF^ groups.

respectively. Notably, _D_DMSN@_M_OMV^PF^ treatment induced the largest enhancement of effector memory T cell among all treatments, suggesting its potential role in providing long-term therapeutic benefits (Fig. 7b, Figure S30 and Figure S31). Immune memory generation can effectively protect the mice from tumor rechallenge. After 40 days of treatment, B16F1 cells were inoculated subcutaneously in the mice (Fig. 7a) and tumor growth was further monitored. As shown in Fig. 7c and d and Figure S32, the tumor growth was most effectively inhibited in _D_DMSN@_M_OMV^PF^ group. Taken together, these results demonstrate that _D_DMSN@_M_OMV^PF^ can induce long-term immune memory and effectively suppress the tumor recurrence.

## 4 Conclusion

In summary, an *in situ* vaccine based on TAA capture was designed to enhance antigen presentation, and activate anti-tumor T cells responses for effective cancer immunotherapy. In this study, the _D_DMSN@_M_OMV^PF^ nanomotor system responded to acidic TME to impair OMV membrane by pH-responsive membrane-disruptive peptide, releasing FA-attached, MTO-embedded OMV fragments. The *in vitro* results demonstrated that tumor cells then could specifically take up these exfoliated fragments due to the tumor-targeting specificity of FA and elicit ICD by the MTO, resulting in DNA-associated TAA release. Subsequently, the exposed DNase propelled _D_DMSN@_M_OMV^PF^ chemotactically moving toward subtle DNA gradients that generated from dying tumor cells, enabling rapid and efficient capture and enrich of TAAs and promoting DCs maturation. This dynamic capture mechanism overcomes the limitations of traditional passive diffusion nanoparticles with low TAA capture efficiency and susceptibility to microenvironment interference, significantly prolonging TAA retention at the tumor site and creating favorable conditions for DC recognition and antigen presentation. The *in vivo* results demonstrated that the _D_DMSN@_M_OMV^PF^ system not only inhibited primary tumor growth but also suppressed distant tumors and prevented recurrence by activating the systemic immune memory effect, which is particularly important in personalized therapy. While advanced strategies such as lymph-node-targeting delivery systems and STING agonist-based therapies represent significant progress, they often face challenges related to efficient *in situ* antigen capture and concentration [[51], [52], [53], [54]]. Compared with existing *in situ* cancer vaccine strategies, this study for the first time combined the dynamic motility of nanomotors with the natural immune activation properties of OMV to achieve a cascade amplification effect of TAA release, dynamic capture and synergies with adjuvants. By spatiotemporally coupling antigen capture with adjuvant delivery, _D_DMSN@_M_OMV^PF^ not only amplifies the immunogenicity of ICD but also establishes durable antitumor immune memory. More importantly, the system does not require exogenous antigen loading or complex personalized preparation, and directly utilizes the tumor endogenous antigens, providing a universal platform for clinical transformation. Overall, this study highlights the promising role of DNase-powered nanomotors for efficient TAAs enrichment and underscores the great potential of _D_DMSN@_M_OMV^PF^ as a novel *in situ* vaccine-like therapeutic system for cancer immunotherapy.

## CRediT authorship contribution statement

**Panpan Song:** Writing – original draft, Visualization, Methodology, Investigation, Formal analysis, Conceptualization. **Xiaoqing Han:** Methodology. **Yanjing Wang:** Methodology. **Xingbo Wang:** Resources. **Yaqing Kang:** Methodology. **Jiao Yan:** Visualization. **Haiyuan Zhang:** Writing – review & editing, Supervision, Resources, Funding acquisition.

## Declaration of competing interest

The authors declare that they have no known competing financial interests or personal relationships that could have appeared to influence the work reported in this paper.

## Data Availability

The data that support the findings of this study are available from the corresponding author upon reasonable request.
