# Supplementary material for: Programmable nanomotor system responsively and chemotactically captures tumor associated antigens for enhanced in situ cancer vaccine
Source: Mater Today Bio. 2025 Nov 11;35:102543. doi: 10.1016/j.mtbio.2025.102543 (PMC12664434; doi:10.1016/j.mtbio.2025.102543)
Supplement: Multimedia component 1 [file mmc1.pdf]

Supplementary Material

Programmable nanomotor system responsively and chemotactically captures tumor associated antigens for enhanced in situ cancer vaccine

Panpan Song [a,c,d], *Xiaoqing Han* [c], *Yanjing Wang* [a,c,d], *Xingbo Wang* [b], *Yaqing Kang* [c], *Jiao Yan* [a,b]* and *Haiyuan Zhang* [a,b]*

[a] The First Affiliated Hospital of Guangzhou Medical University, Guangzhou Medical University, Guangzhou, 510120, China

[b] School of Biomedical Engineering, Guangzhou Medical University, Guangzhou, 511436, China

[c] Changchun Institute of Applied Chemistry, Chinese Academy of Sciences, Changchun,130022, China

[d] School of Applied Chemistry and Engineering, University of Science and Technology of China, Hefei, 230026, China

*Corresponding authors:

Haiyuan Zhang, E-mail: hzhang@gzhmu.edu.cn

Jiao Yan, E-mail: yanjiao@gzhmu.edu.cn

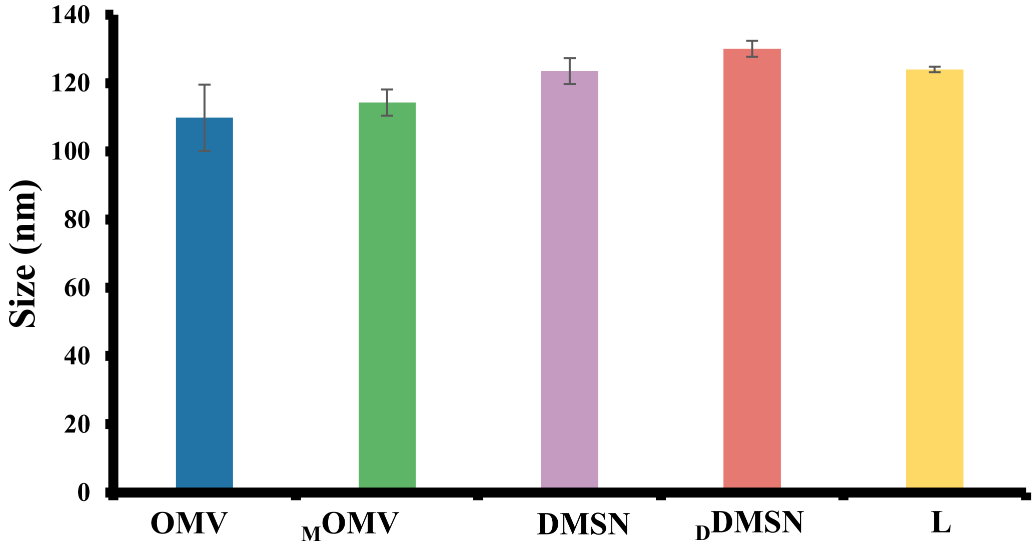

Figure S1. Hydrodynamic sizes of OMV, $_{M}$OMV, DMSN, $_{D}$DMSN and L(n=3).

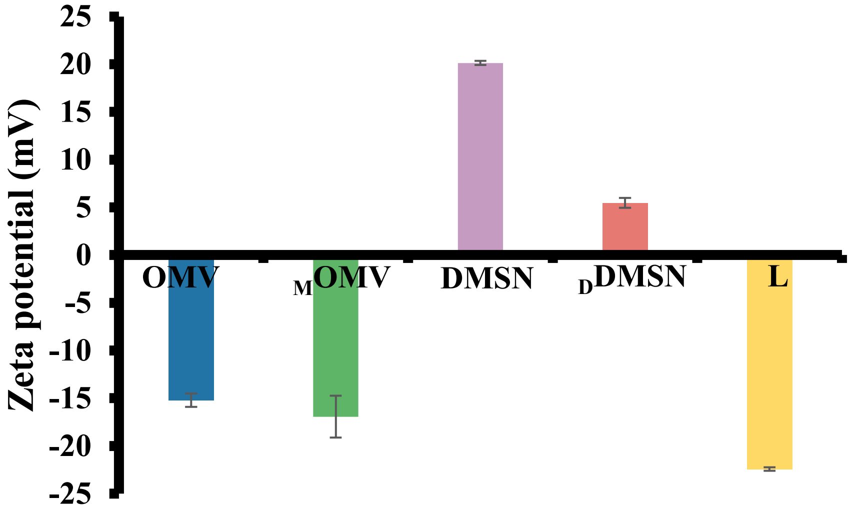

Figure S2. Zeta potentials of OMV, $_{M}$OMV, DMSN, $_{D}$DMSN and L(n=3).

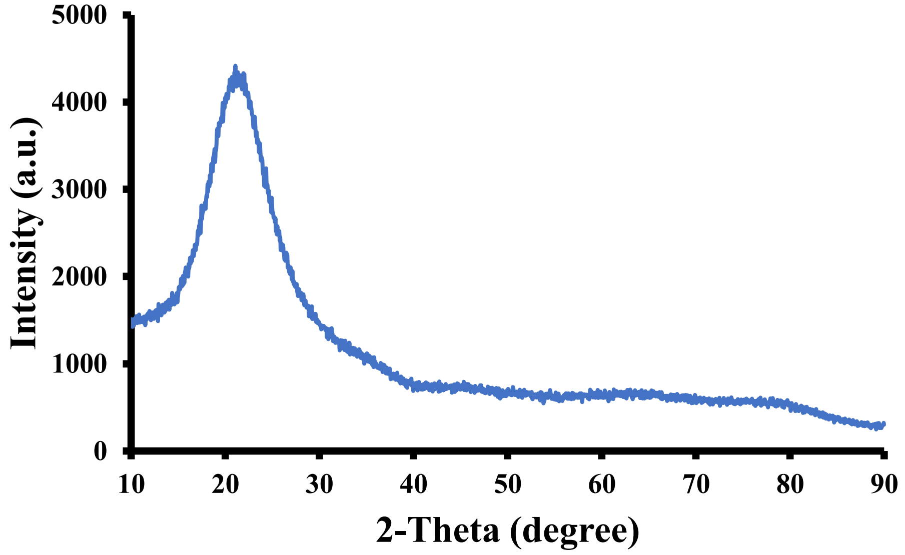

Figure S3. XRD diffraction pattern of DMSN.

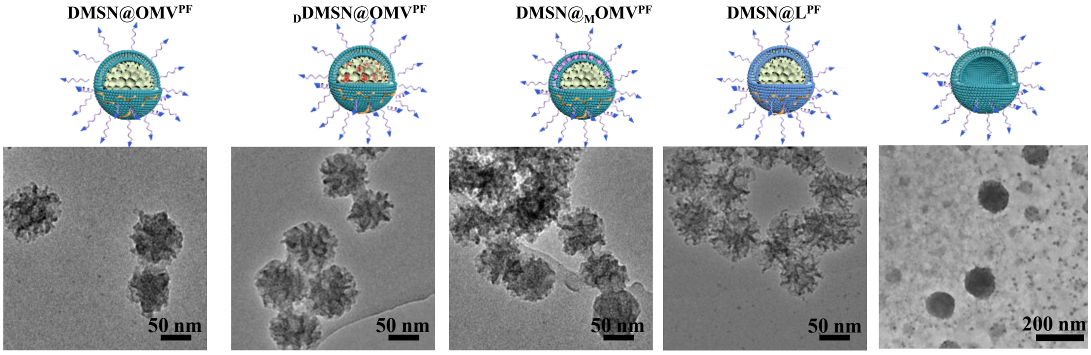

Figure S4. TEM images of DMSN@OMV^{PF}, $_D$DMSN@OMV^{PF}, DMSN@$_M$OMV^{PF}, DMSN@L^{PF} and L.

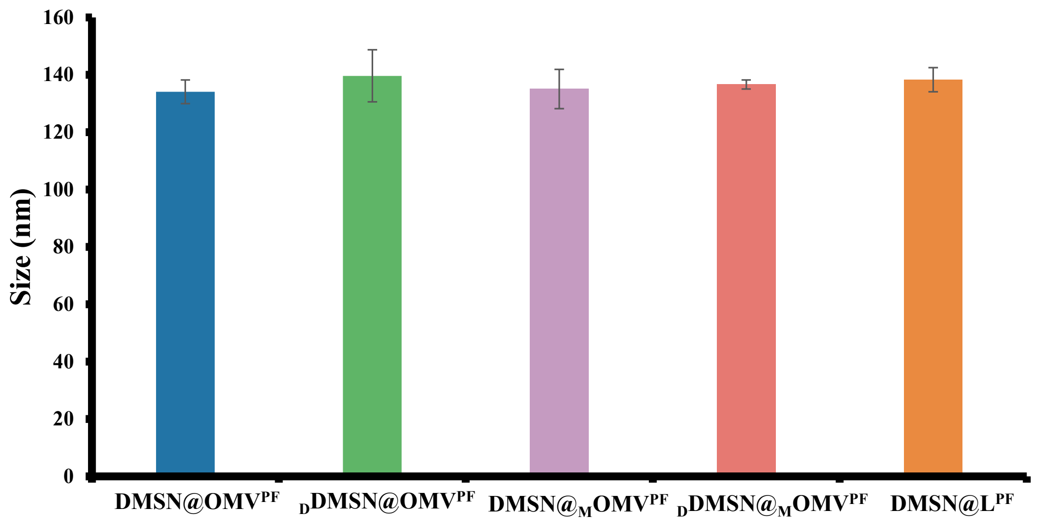

Figure S5. Hydrodynamic size of DMSN@OMV^{PF}, $_{D}$DMSN@OMV^{PF}, DMSN@$_{M}$OMV^{PF}, $_{D}$DMSN@$_{M}$OMV^{PF} and DMSN@L^{PF} in DI water (n=3).

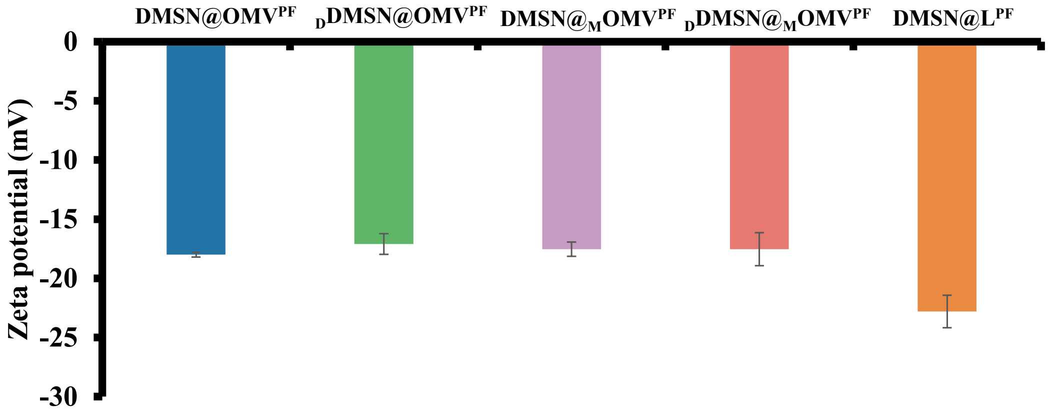

Figure S6. Zeta potentials of DMSN@OMV^{PF}, $_{D}$DMSN@OMV^{PF}, DMSN@$_{M}$OMV^{PF}, $_{D}$DMSN@$_{M}$OMV^{PF} and DMSN@L^{PF} in DI water (n=3).

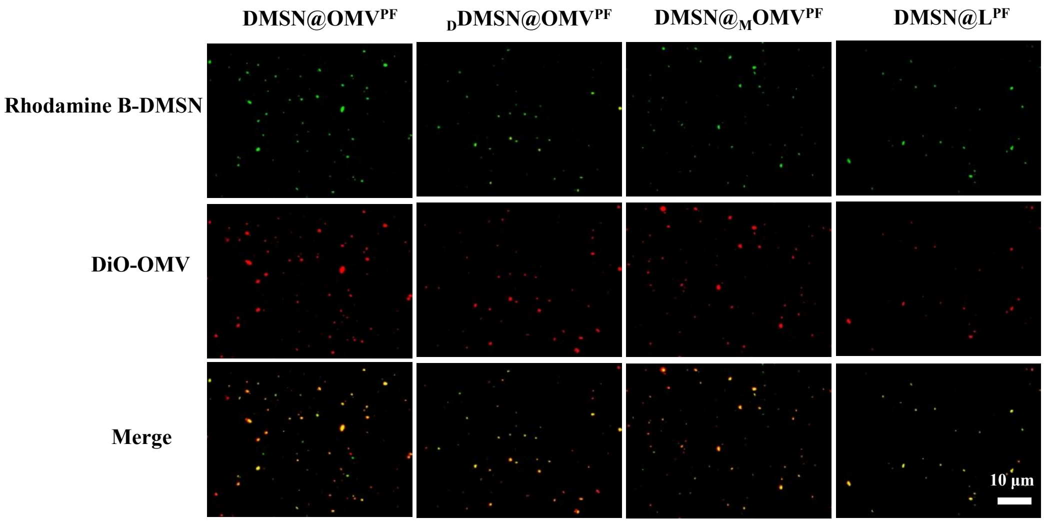

Figure S7. CLSM images of DMSN@OMV^{PF}, $_{D}$DMSN@OMV^{PF}, DMSN@$_{M}$OMV^{PF} and DMSN@L^{PF}. OMV was labeled with lipophilic dye DiO (green) while DMSN with Rhodamine B.

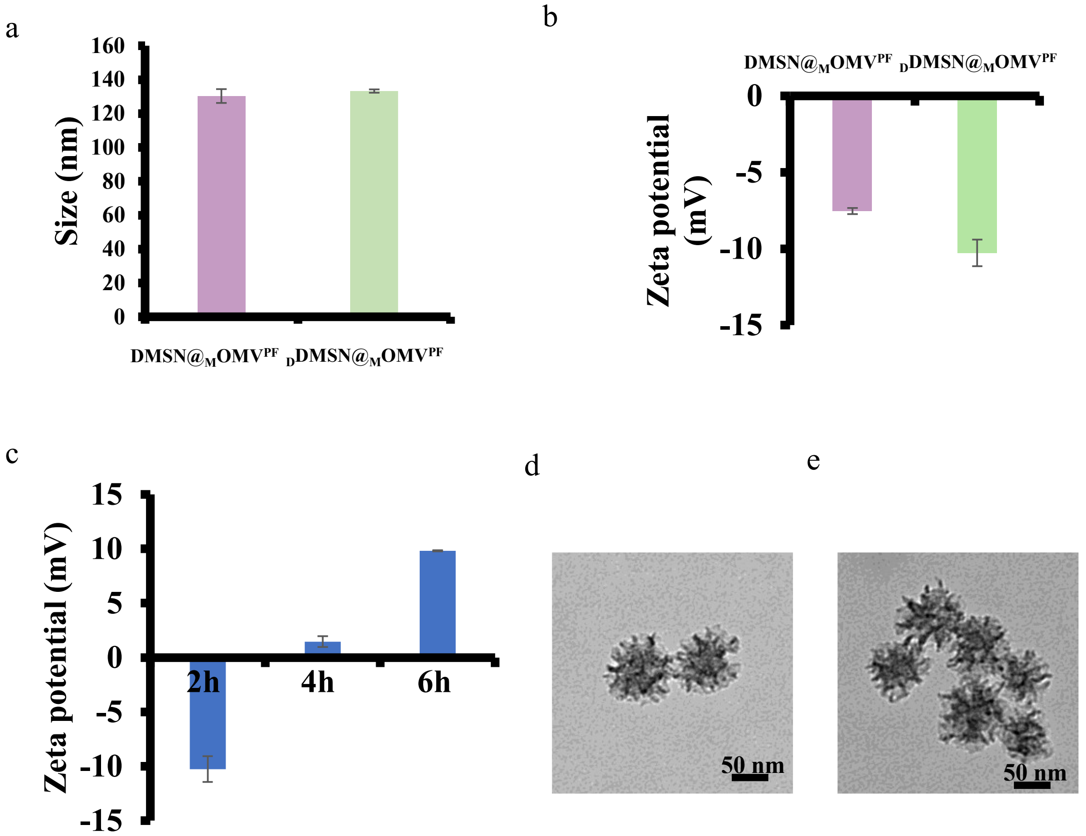

Figure S8 (a) Hydrodynamic diameter and (b) zeta potential of DMSN@$_{M}$OMV^{PF} and $_{D}$DMSN@$_{M}$OMV^{PF} at pH 6.5; (c) Zeta potential of $_{D}$DMSN@$_{M}$OMV^{PF} incubated in PBS (pH 6.5) containing 2.5 μmol L^{-1} DNA for various time periods; (d) TEM of DMSN@$_{M}$OMV^{PF} and (e) $_{D}$DMSN@$_{M}$OMV^{PF} at pH 6.5.

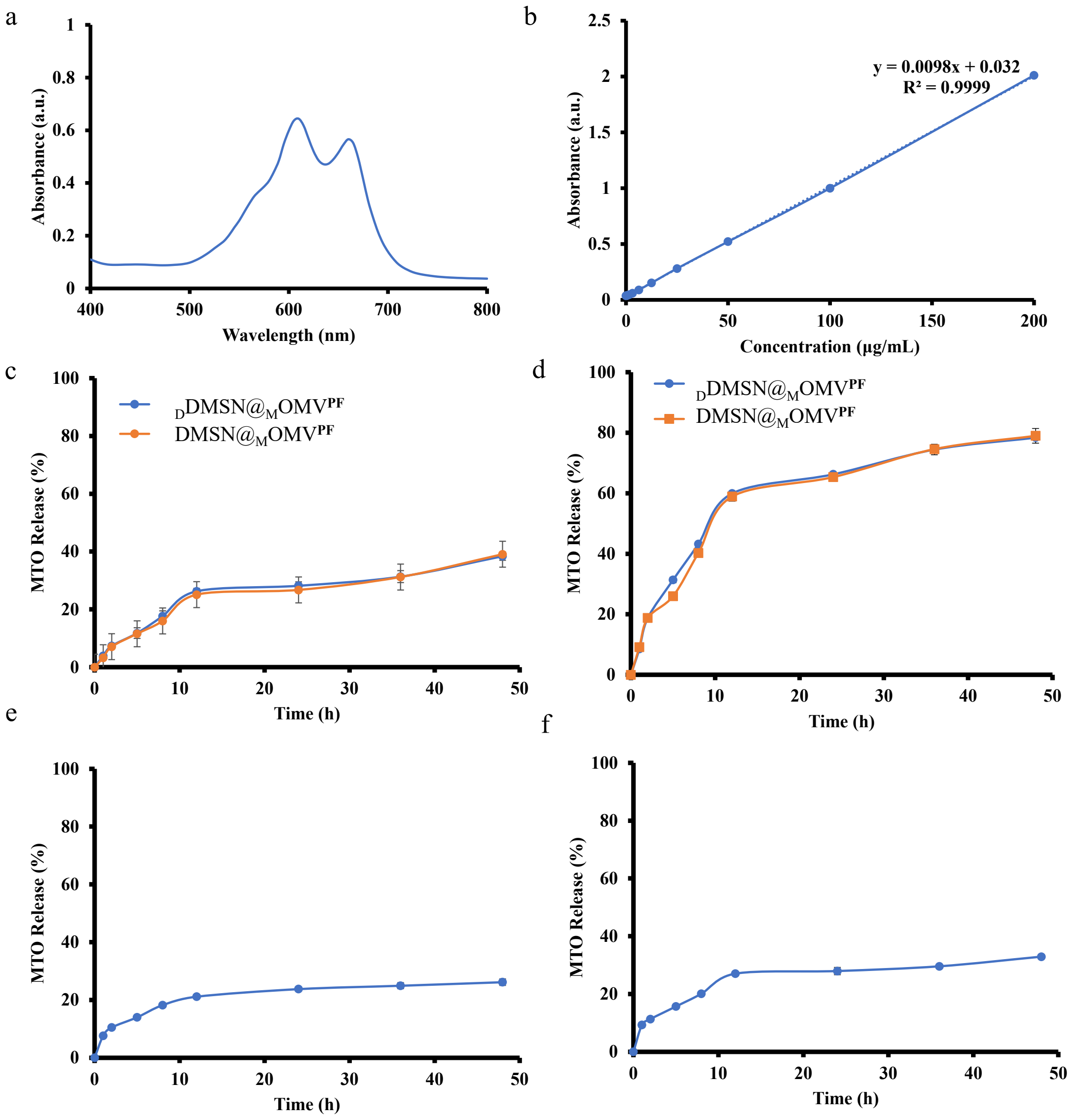

Figure S9 (a) UV-vis absorption spectrum of MTO and (b) the corresponding standard curve and linear regression equation; (c) MTO release profiles from DMSN@$_M$OMV^{PF} and $_D$DMSN@$_M$OMV^{PF} at pH 7.4 and (d) pH 6.5; (e) MTO release profiles from DMSN@$_M$OMV^F at pH 7.4 and (f) pH 6.5.

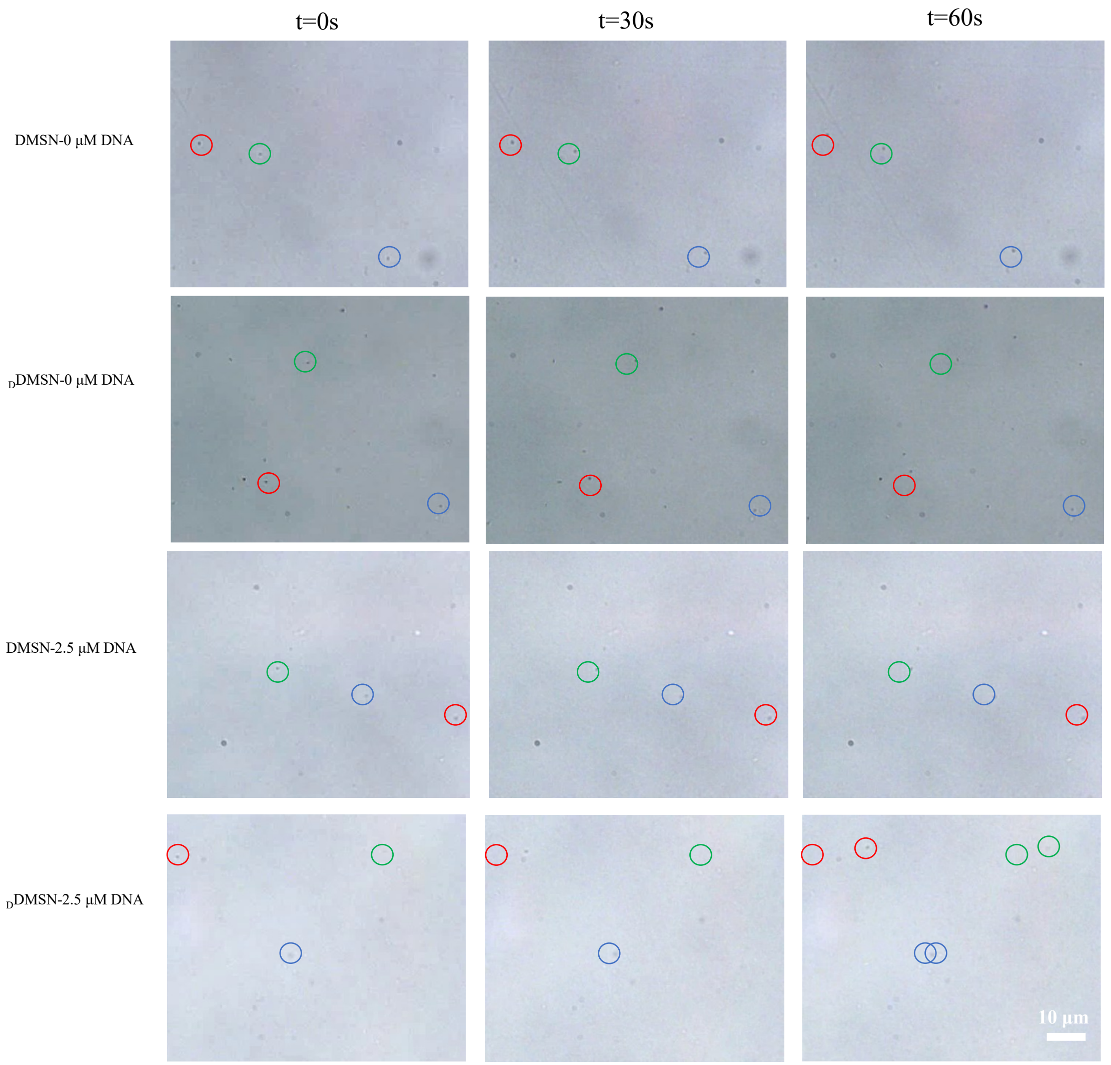

Figure S10. Microscopic images of DDMSN and DMSN in the presence or absence of DNA at different time points.

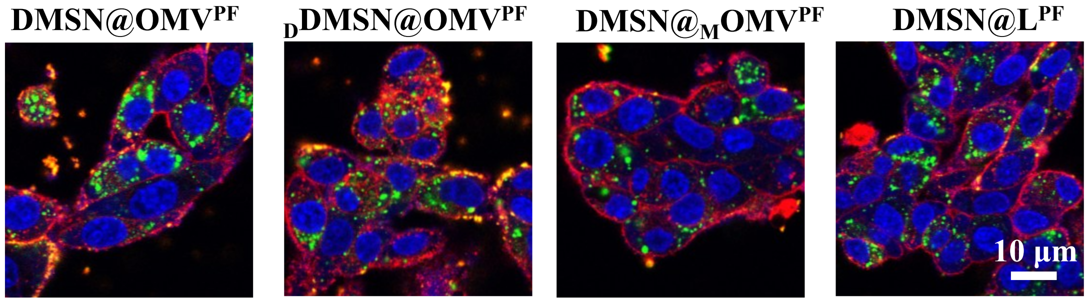

Figure S11. CLSM image analysis of OMV fragment uptake by B16F1 cells treated with DiO-labeled DMSN@OMV^{PF}, $_{D}$DMSN@OMV^{PF}, DMSN@$_{M}$OMV^{PF} and DMSN@L^{PF} NPs.

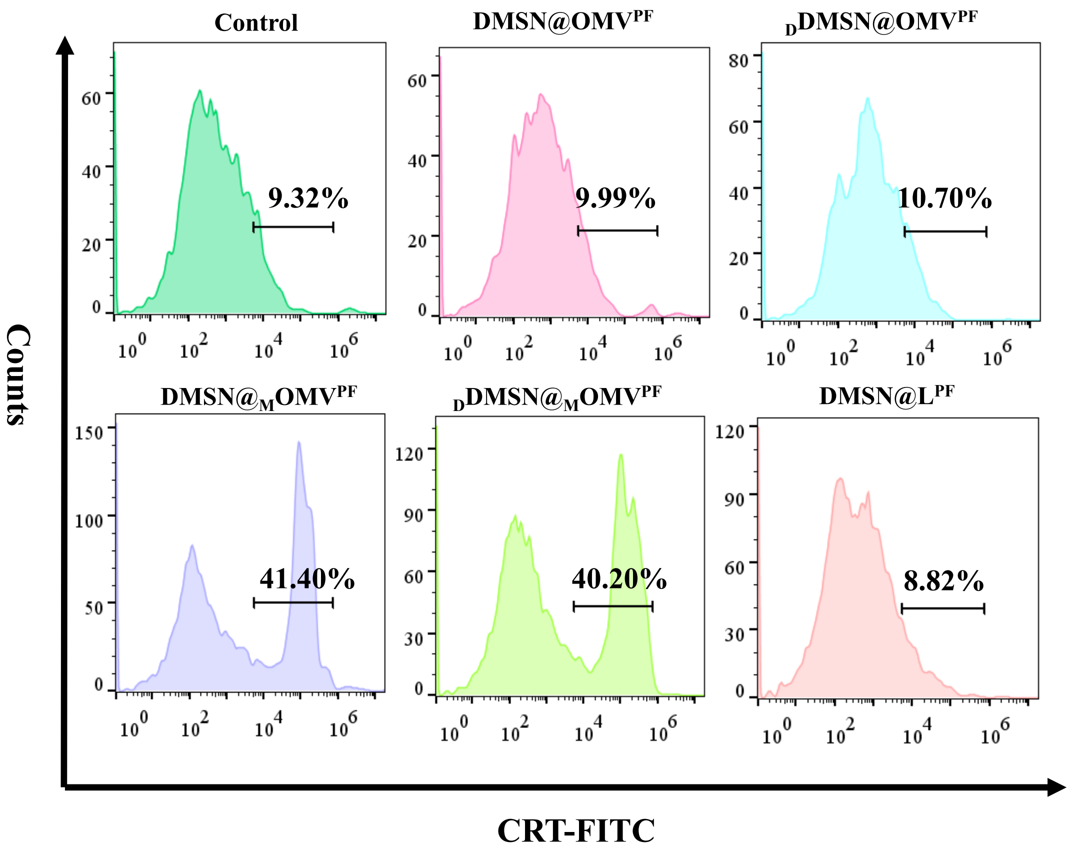

Figure S12. Percentages of live CRT-positive B16F1 cells (CRT^{+}/PI^{-}) after treated with PBS, DMSN@OMV^{PF}, $_{D}$DMSN@OMV^{PF}, DMSN@$_{M}$OMV^{PF}, $_{D}$DMSN@$_{M}$OMV^{PF} and DMSN@L^{PF} NPs (equivalent to 20 μg mL^{-1} DMSN) for 24 h.

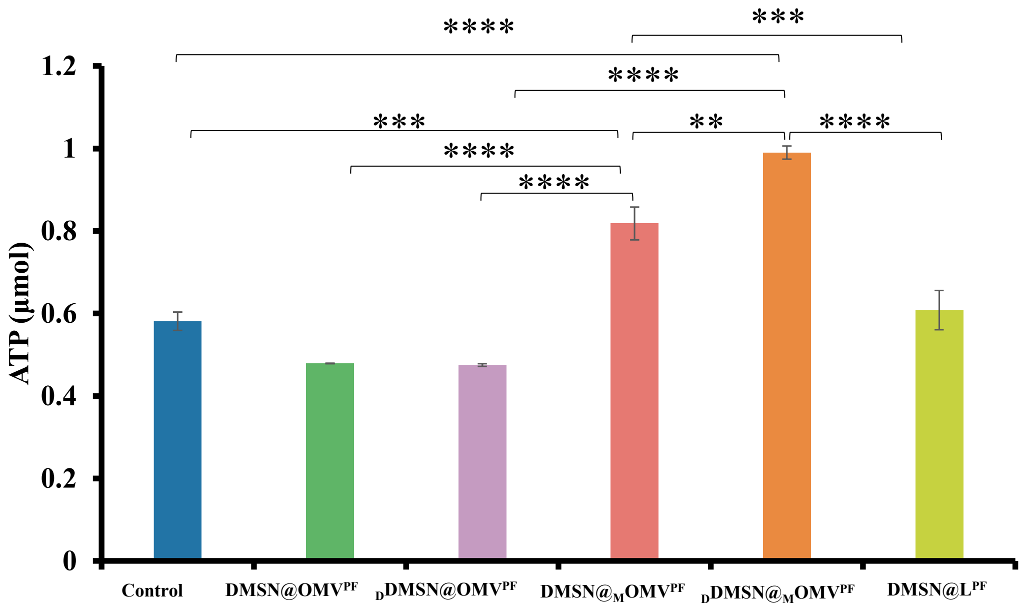

Figure S13. ATP release from B16F1 cells after treated with PBS, DMSN@OMV^{PF}, $_{D}$DMSN@OMV^{PF}, DMSN@$_{M}$OMV^{PF}, $_{D}$DMSN@$_{M}$OMV^{PF} and DMSN@L^{PF} NPs (equivalent to 20 µg mL^{-1} DMSN) for 24 h. ns: not significant (p>0.05), *: $p < 0.05$, **: $p < 0.01$, ***: $p < 0.001$, ****: $p < 0.0001$.

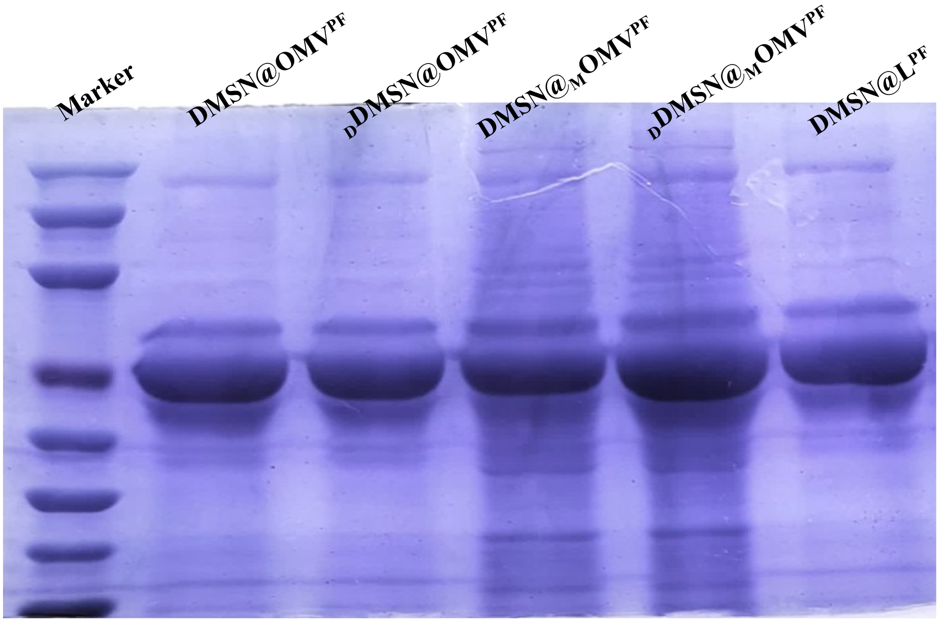

Figure S14. SDS-PAGE analysis of DMSN@OMV^{PF}, $_{D}$DMSN@OMV^{PF}, DMSN@$_{M}$OMV^{PF}, $_{D}$DMSN@$_{M}$OMV^{PF} and DMSN@L^{PF} NPs after incubated with B16F1 cells for 24 h.

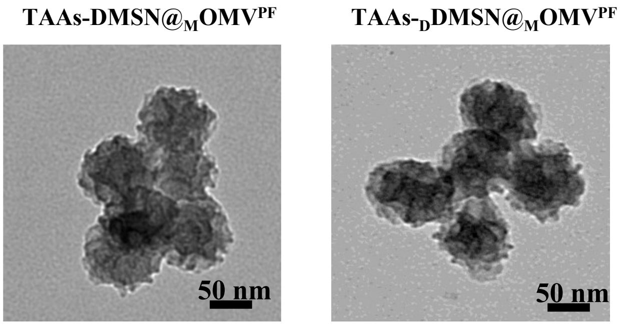

Figure S15. TEM of TAAs captured DMSN@$_M$OMV^{PF} and $_D$DMSN@$_M$OMV^{PF}.

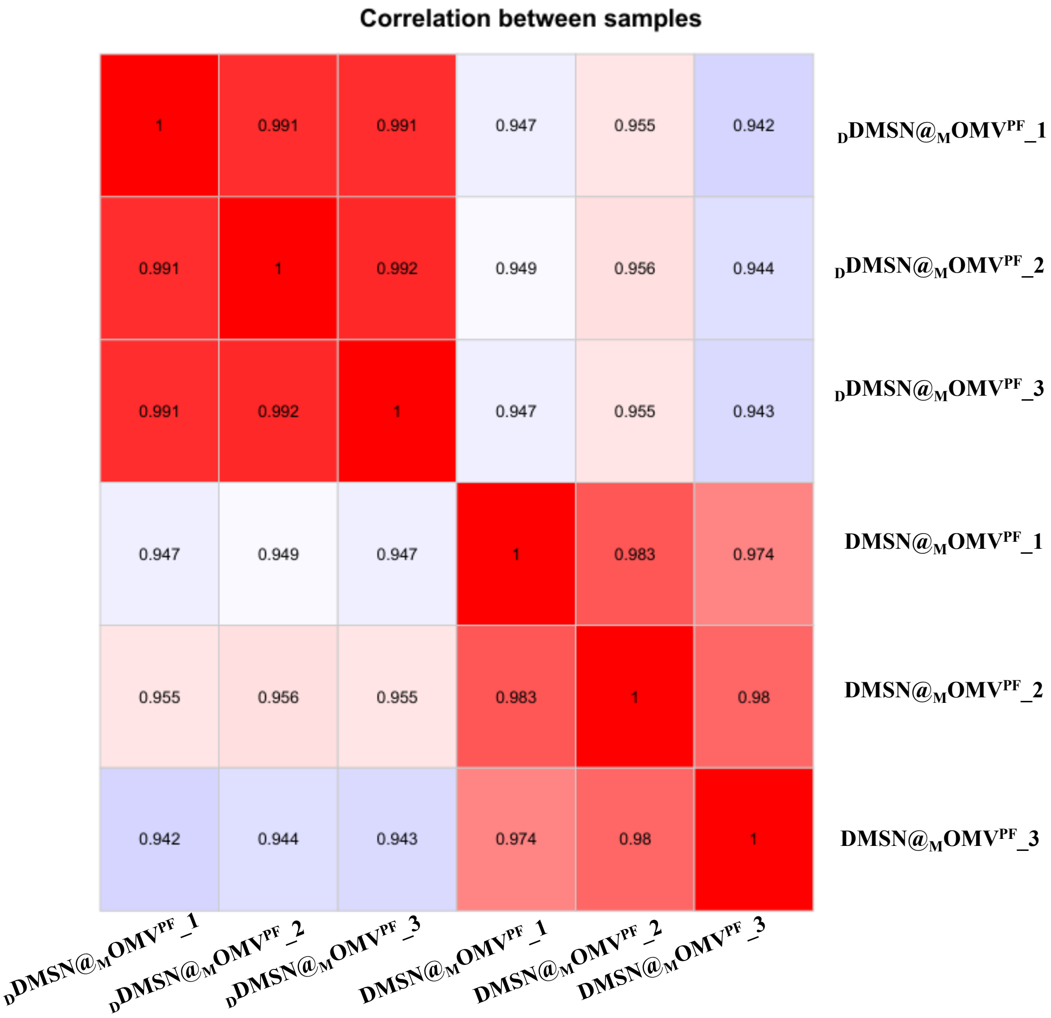

Figure S16. Correlation analysis on the captured total protein samples.

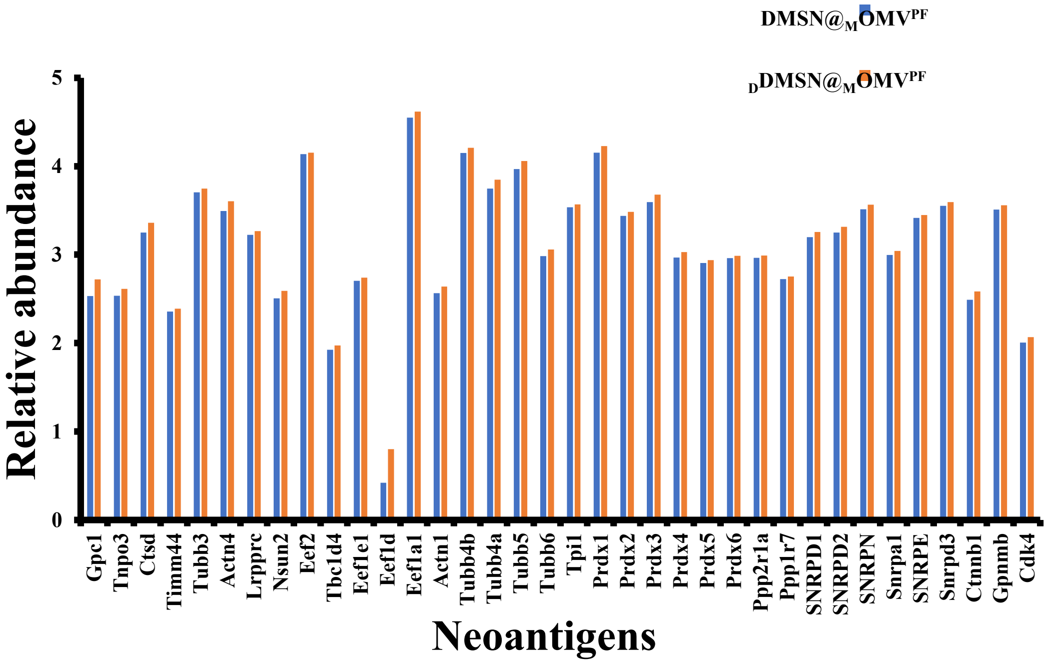

Figure S17. The relative abundance of neoantigens captured by DMSN@$_{M}$OMV^{PF} and $_{D}$DMSN@$_{M}$OMV^{PF}.

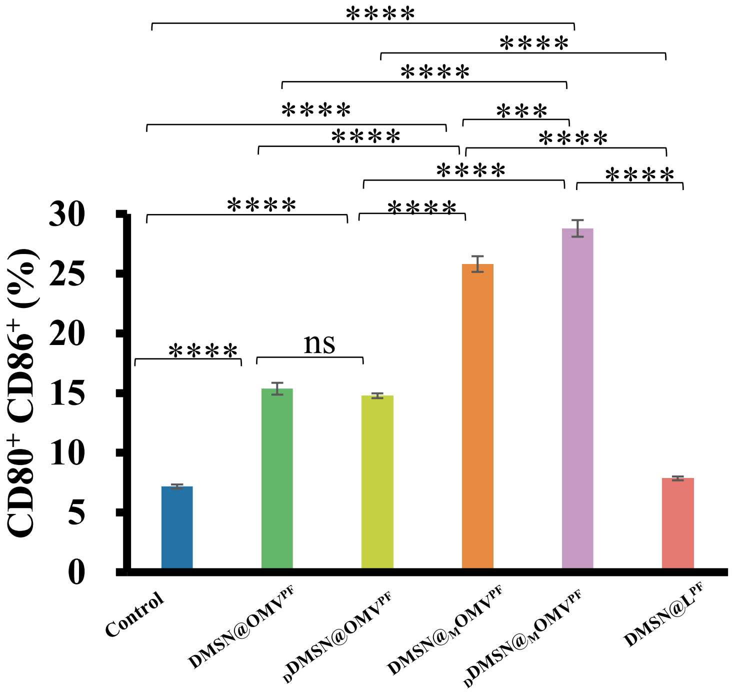

Figure S18. Quantitative analysis of CD80^{+} CD86^{+} level in Figure 4h.

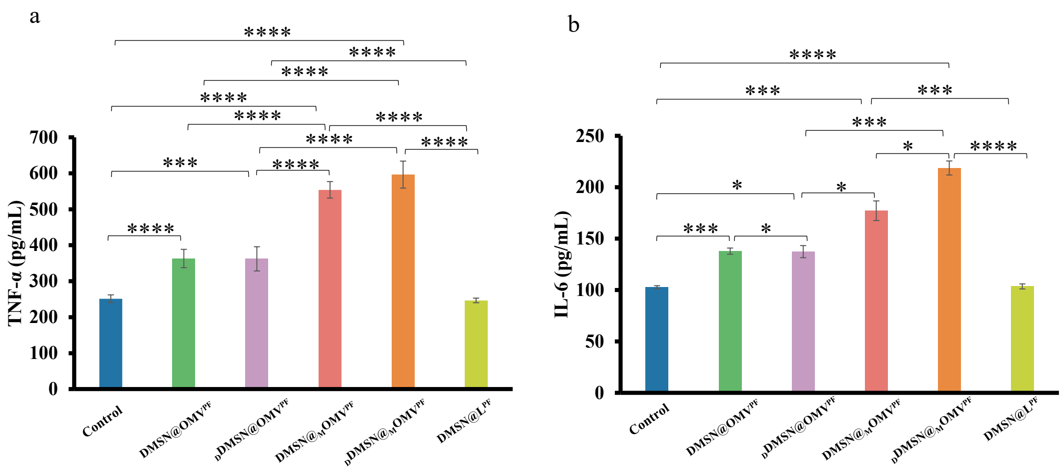

Figure S19. Cytokine levels of TNF-α (a) and IL-6 (b) in the supernatant of BMDCs culture as measured by ELISA (n=3), ns: not significant (p>0.05), *: $p < 0.05$, **: $p < 0.01$, ***: $p < 0.001$, ****: $p < 0.0001$.

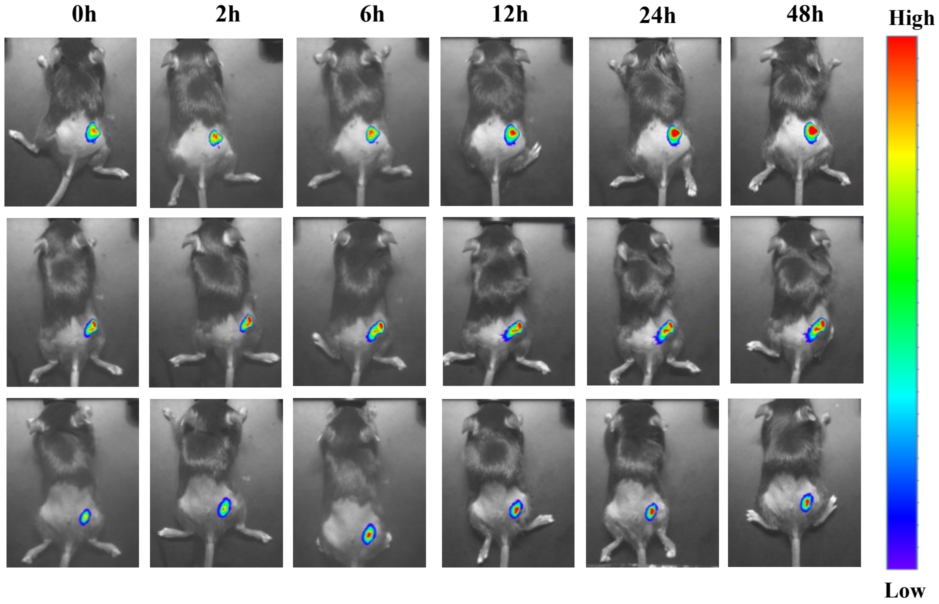

Figure S20. Representative fluorescence images of Cy5.5-labeled $_{D}$DMSN@$_{M}$OMV^{PF} -treated mice *in vivo* at different time points (n=3).

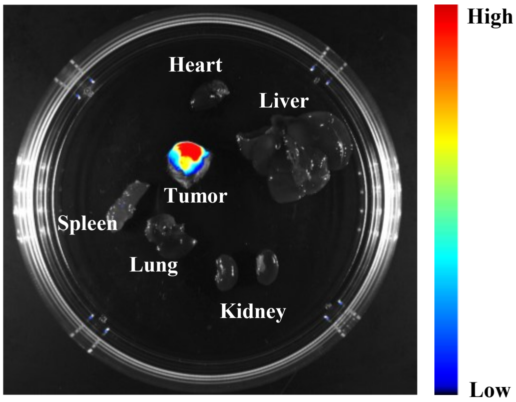

Figure S21. Fluorescence images of major organs and tumors at 48 h-post injection of Cy5.5-labeled $_{D}$DMSN@$_{M}$OMV^{PF}.

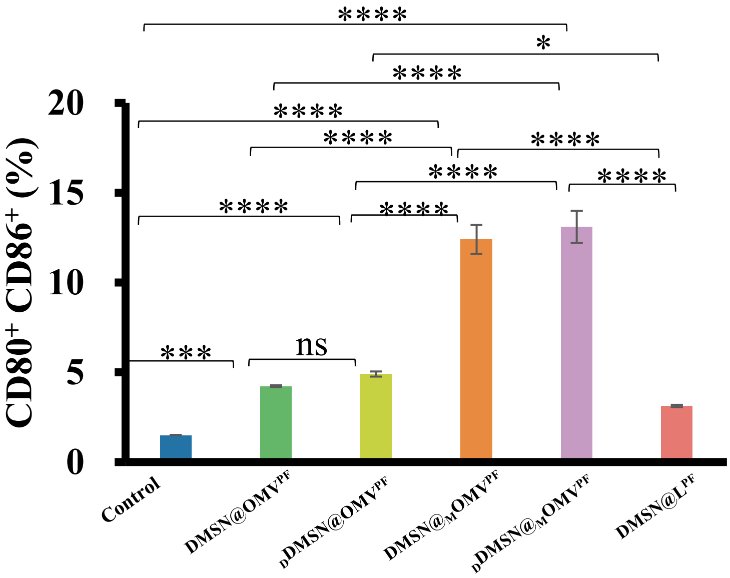

Figure S22. Quantitative analysis of CD80^{+} CD86^{+} level in Figure 5d.

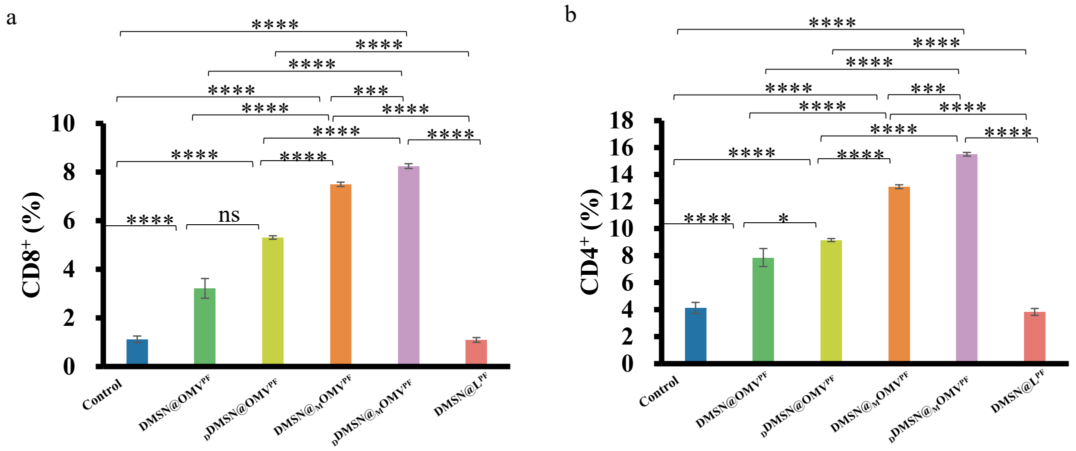

Figure S23. Quantitative analysis of $CD8^+$ (a) and $CD4^+$ (b) level in Figure 5e.

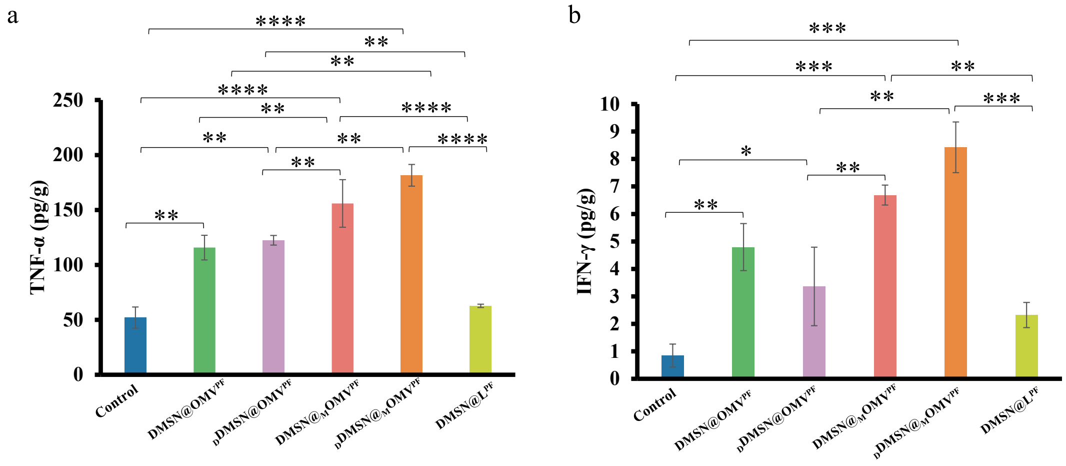

Figure S24. Cytokine levels of TNF-α (a) and IFN-γ (b) in various tumor extracts isolated after treatment as measured by ELISA (n=5), ns: not significant (p>0.05), *: $p < 0.05$, **: $p < 0.01$, ***: $p < 0.001$, ****: $p < 0.0001$.

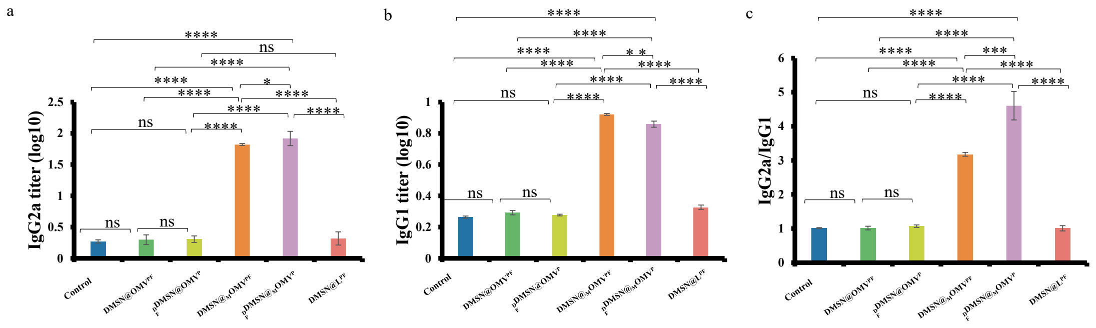

Figure S25. Expression of serum antibody of (a) IgG1 and (b) IgG2a of treated mice; (c) The ratio of IgG2a to IgG1.

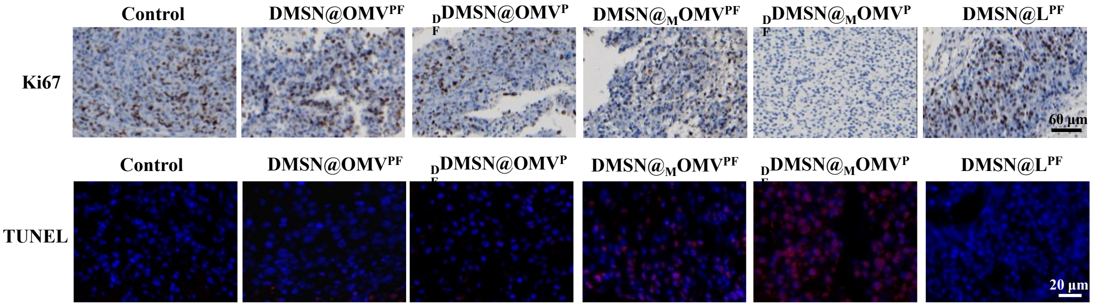

Figure S26. Ki-67 immunohistochemistry staining and TUNEL staining of tumors in different treatment groups.

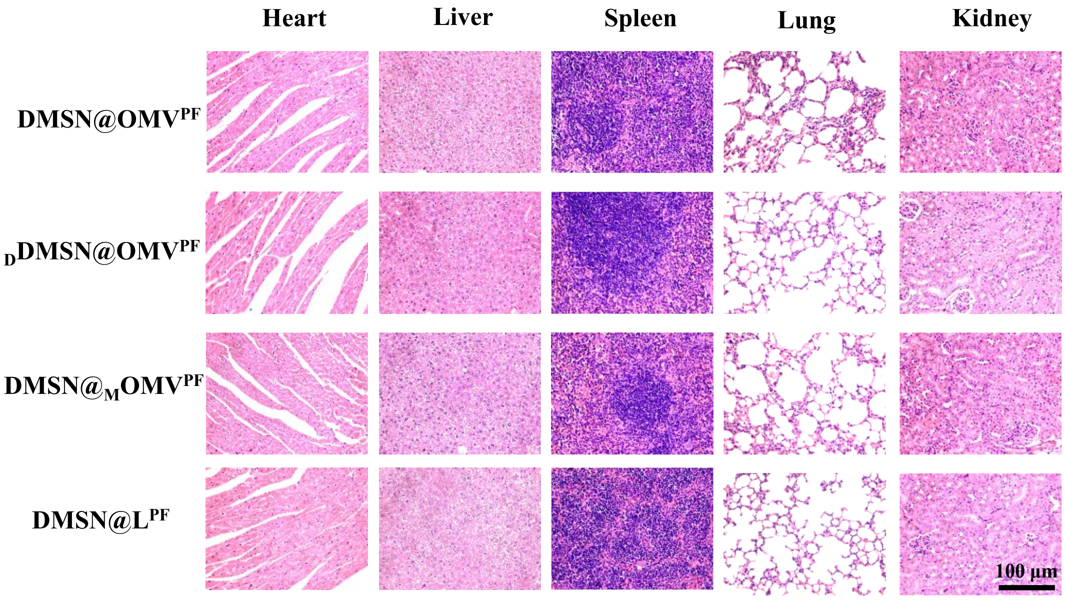

Figure S27. H&E staining of major organs of B16F1 tumor-bearing mice after treated with DMSN@OMV^{PF}, $_{D}$DMSN@OMV^{PF}, DMSN@$_{M}$OMV^{PF} and DMSN@L^{PF} NPs for 16 days.

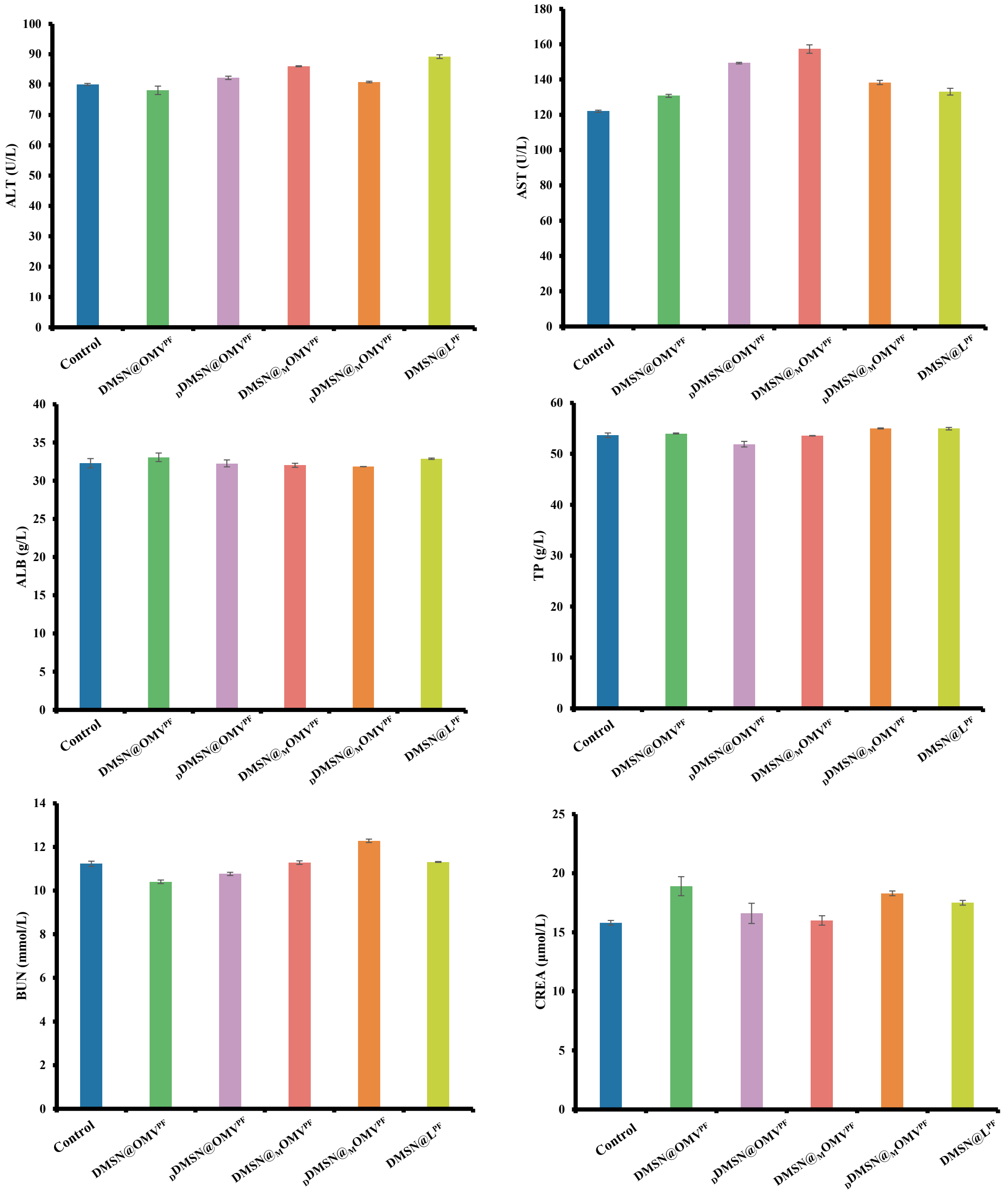

Figure S28. Blood biochemistry of B16F1 tumor-bearing C57BL/6 mice after treated with PBS, DMSN@OMV^{PF}, $_{D}$DMSN@OMV^{PF}, DMSN@$_{M}$OMV^{PF}, $_{D}$DMSN@$_{M}$OMV^{PF} and DMSN@L^{PF} NPs for 16 days. The range of parameters of normal mice were referenced from the website of Charles River (www.criver.com): alanine transaminase (ALT): (27-195) U/L, aspartate transamine (AST): (43-397) U/L, albumin (ALB): (24-43) g/L, total protein (TP): (48-72) g/L, blood urea (BUN): (5.0-26) mmol/L, and creatinine (CREA): (11-28) μmol/L.

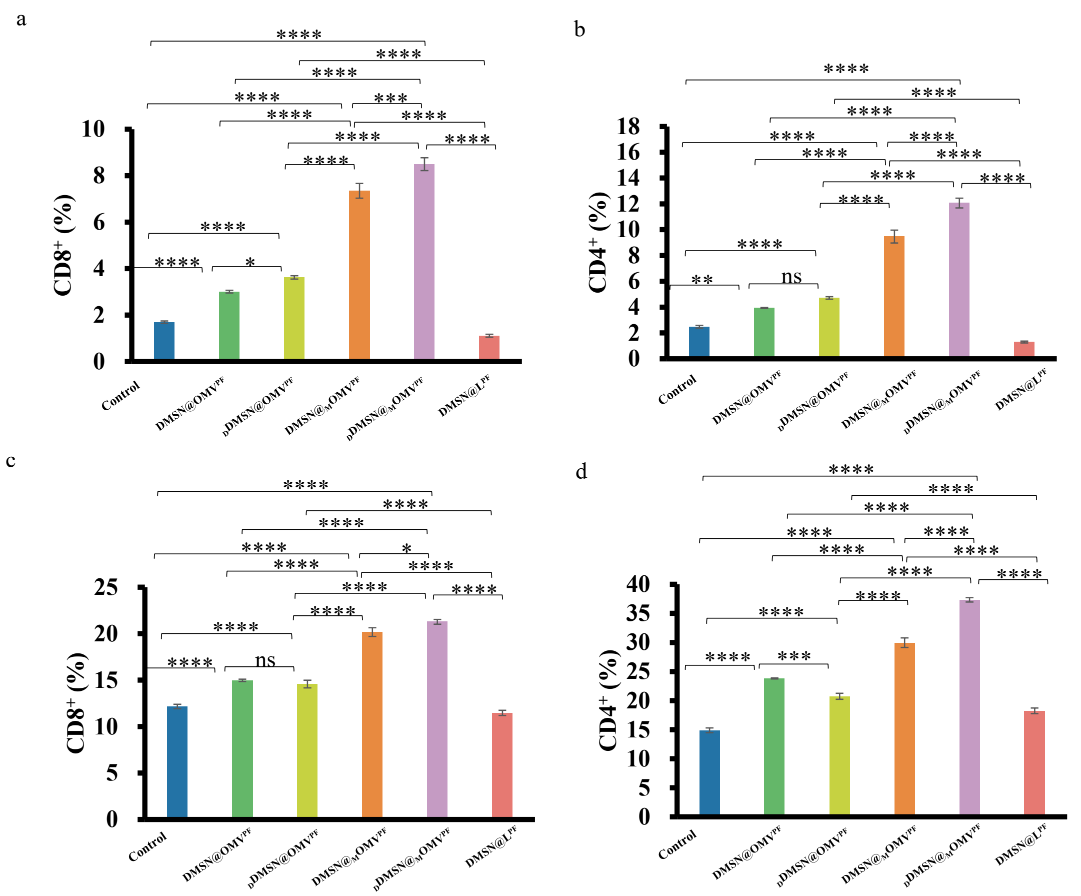

Figure S29. Quantitative analysis of $CD8^+$ (a) (c) and $CD4^+$ (b) (d) levels in Figure 6g and 6h.

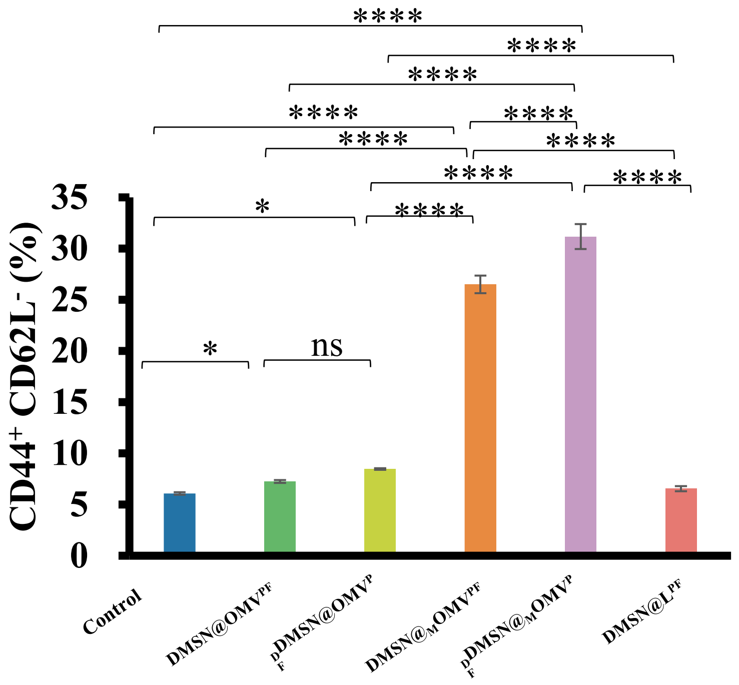

Figure S30. Quantitative analysis of CD44^{+} CD62L^{-} levels in Figure 7b.

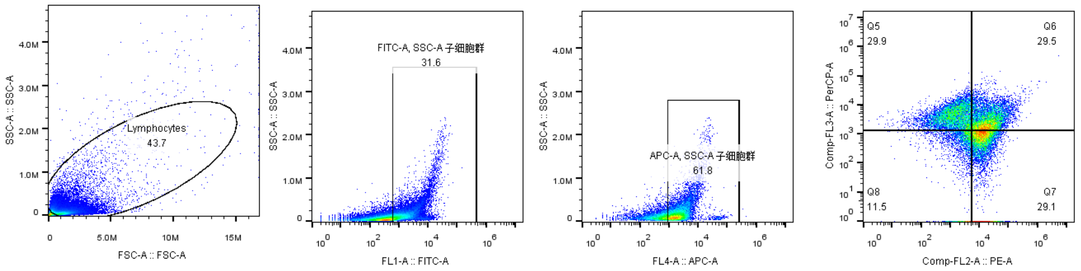

Figure S31. The complete original gating strategy for the flow cytometry analysis of $_{D}$DMSN@$_{M}$OMV^{PF} in Figure 7b.

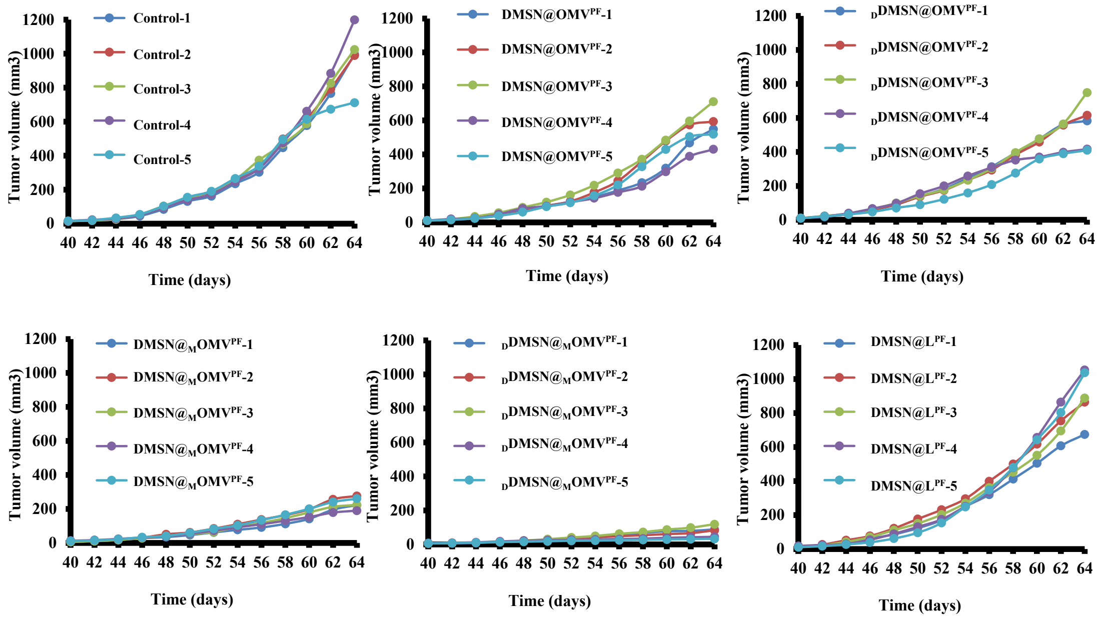

Figure S32. Individual tumor growth curves of tumor rechallenge mice model after various treatments.
